# Early best available antibiotic therapy for multidrug-resistant gram-negative bloodstream infections: a target trial emulation

**DOI:** 10.1016/j.eclinm.2026.104302

**Published:** 2026-10-29

**Authors:** Luca Mezzadri, Wei Cong Tan, Xinxin Hao, Joan Quah, Abi Manesh, Ya-Ting Chang, Anh T.K. Nguyen, Ying Ding, Suwatthiya Kitsaran, Hock Hin Chua, Sotharith Bory, Sasheela Ponnampalavanar, Siriluck Anunnatsiri, Mohd Zulfakar Mazlan, Azizullah Khan Dhiloo, Rongpong Plongla, Andrea Lay-Hoon Kwa, Syed Faisal Mahmood, Arthur Dessi Roman, C Louise Thwaites, Phan Vinh Tho, Sheng Wu, Darunee Chotiprasitsakul, Ke Juin Wong, Hendri Wijaya, Cybele L. Abad, Muhammad Osama Rehman Khalid, Gazi Md. Salahuddin Mamun, Giri Shan Rajahram, Helio Gutierres, Huynh Ngoc Hon, Tran Van Giang, Ngoc Thach Pham, Abhilasha Karkey, Samanmalee Gunasekara, Raph L. Hamers, Robert Sinto, Inke N.D. Lubis, Minggui Wang, Merlin Moni, Po-Yu Liu, Yonghong Xiao, Basudha Khanal, N. Lakshmi Priya, Yohei Doi, Hitoshi Honda, Bayaraa Baljin, Ulziijargal Gurav, Rosmonaliza Asli, Lowell Ling, Ashesh Dhungana, Li-Yang Hsu, David L. Paterson, Yin Mo, ACORN-HAI investigators, Jie Su, May Gee Ong, Kee Mee Tan, Amanda Wei Ping Ling, Shweta Rajkumar Singh, Siew Pei Ong, Jia Rhou Lai, Shiau Hui Diong, Suchart Booraphun, Ratiporn Bansong, Xun Ting Tiong, Alisa Hanum Arba'eni, Sophany Koy, Seanghuoy Ho, Helmi Bin Sulaiman, Soo Nee Tang, Pope Kosalaraksa, Zeti Norfidiyati Salmuna, Gabriel Zherong Yan, Ambreen Fatima, Suvaporn Anugulruengkitt, Sadia Shakoor, Cecilia C. Maramba Lazarte, Thi Kim Anh Nguyen, Jinlan Lin, Pitak Santanirand, Ruth Tsin En Cheng, Rina Yunita, Nicole Bernadette Terre, Madiha Hashmi, Md. Ali Amin Nabin, Mohd Zaki Bin Mohd Zaili, Joshua Francis, H. Rogier van Doorn, Bidhya Sharma Sharma, Sumudu Withanage, Yulia R. Saharman, R. Lia Kusumawati Kusumawati, Xiaohua Qin, Chithra Tomy, Chien-Hao Tseng, Ping Shen, Shyam Prasad Kafle, T. Palaniappan, Shogo Hanai, Damdindorj Boldbaatar, Mohamed Ibrahim Ali Alsaman, Khurelbaatar Nyamdavaa, Damdindorj Boldbaatar, Christopher Lai, Santosh Poudel

**Affiliations:** a ADVANCE-ID, Saw Swee Hock School of Public Health, National University of Singapore, Singapore, Singapore; b School of Medicine and Surgery, University of Milano-Bicocca, Milan, Italy; c Division of Infectious Diseases, University Medicine Cluster, National University Hospital, Singapore, Singapore; d Department of Medicine, National University of Singapore, Singapore, Singapore; e Department of Pharmacy, National University Hospital, Singapore, Singapore; f Department of Infectious Diseases, Christian Medical College, Vellore, Tamil Nadu, India; g Division of Infectious Diseases, Department of Internal Medicine, Kaohsiung Medical University Hospital, Kaohsiung Medical University, Kaohsiung, Taiwan; h Oxford University Clinical Research Unit (OUCRU), Ho Chi Minh City, Vietnam; i Hospital for Tropical Diseases, Ho Chi Minh City, Vietnam; j Sunpasitthiprasong Hospital, Ubon Ratchathani, Thailand; k Sarawak General Hospital, Sarawak, Malaysia; l Calmette Hospital, Phnom Penh, Cambodia; m Universiti Malaya Medical Centre, Kuala Lumpur, Malaysia; n Division of Infectious Diseases and Tropical Medicine, Faculty of Medicine, Khon Kaen University, Khon Kaen, Thailand; o Department of Anaesthesiology and Intensive Care, School of Medical Sciences, Universiti Sains Malaysia, 16150, Kubang Kerian, Kelantan, Malaysia; p Dow University of Health Sciences, Karachi, Pakistan; q Division of Infectious Diseases, and Center of Excellence in Antimicrobial Resistance and Stewardship, Department of Medicine, Faculty of Medicine, Chulalongkorn University, King Chulalongkorn Memorial Hospital, Thai Red Cross Society, Bangkok, Thailand; r Singapore General Hospital, Pharmacy (Research), Singapore, Singapore; s The Aga Khan University Hospital, Karachi, Pakistan; t Philippine General Hospital, Manila, Philippines; u Hospital Nacional Guido Valadares, Dili, Timor-Leste; v Trung Vuong Hospital, Ho Chi Minh City, Vietnam; w Beijing Tsinghua Changgung Hospital, Beijing, China; x Faculty of Medicine Ramathibodi Hospital, Mahidol University, Bangkok, Thailand; y Sabah Women and Children's Hospital, Kota Kinabalu, Malaysia; z Adam Malik Hospital, Medan, Indonesia; aa Faculty of Medicine, Universitas Sumatera Utara, Medan, Indonesia; ab The Medical City, Metro Manila, Philippines; ac University of the Philippines College of Medicine (UPCM), Manila, Philippines; ad Dr. Ziauddin Hospital Clifton Campus, Karachi, Pakistan; ae International Centre for Diarrhoeal Disease Research, Bangladesh (icddr,b), Dhaka, Bangladesh; af Queen Elizabeth Hospital II, Ministry of Health Malaysia, Kota Kinabalu, Malaysia; ag National Hospital for Tropical Diseases, Hanoi Medical University, Ha Noi, Vietnam; ah Oxford University Clinical Research Unit (OUCRU), Patan Hospital, Kathmandu, Nepal; ai Patan Hospital, Kathmandu, Nepal; aj Bir Hospital, Kathmandu, Nepal; ak National Cancer Institute, Maharagama, Sri Lanka; al Oxford University Clinical Research Unit Indonesia, Faculty of Medicine Universitas Indonesia, Jakarta, Indonesia; am Centre for Tropical Medicine and Global Health, Nuffield Department of Medicine, University of Oxford, Oxford, United Kingdom; an Department of Internal Medicine, Dr Cipto Mangukusumo Hospital, Faculty of Medicine Universitas Indonesia, Jakarta, Indonesia; ao Prof. Chairuddin P. Lubis Universitas Sumatera Utara Hospital, Medan, Indonesia; ap Huashan Hospital Affiliated to Fudan University, Shanghai, China; aq Division of Infectious Diseases, Department of General Medicine, Amrita Hospital, Amrita Viswavidhyapeedam, Kochi, Kerala, India; ar Taichung Veterans General Hospital, Taichung, Taiwan; as The First Affiliated Hospital, Zhejiang University, Hangzhou, China; at B.P. Koirala Institute of Health Sciences, Dharan, Nepal; au Medway Hospitals, Kodambakkam, India; av Department of Infectious Diseases, Fujita Health University School of Medicine, Toyoake, Japan; aw Mongolian National University of Medical Sciences, Ulaanbaatar, Mongolia; ax Raja Isteri Pengiran Anak Saleha Hospital, Bandar Seri Begawan, Brunei Darussalam; ay Department of Anaesthesia and Intensive Care, The Chinese University of Hong Kong, Hong Kong Special Administrative Region of China; az Infectious Diseases Translational Research Programme, Yong Loo Lin School of Medicine, National University of Singapore, Singapore, Singapore; ba Centre for Tropical Medicine, Nuffield Department of Medicine, University of Oxford, Oxford, UK; bb Mahidol-Oxford Tropical Medicine Research Unit, Faculty of Tropical Medicine, Mahidol University, Bangkok, Thailand

**Keywords:** Bloodstream infections, Multidrug-resistant gram-negative bacteria, Antimicrobial resistance, Early appropriate antibiotic therapy, Target trial emulation

## Abstract

**Background:**

The effect of timely appropriate antibiotics in multidrug-resistant Gram-negative bacteria (MDR-GNB) bloodstream infections (BSIs) remains uncertain. Robust evidence is needed to inform diagnostic and treatment strategies, especially across settings with high antimicrobial resistance (AMR) and varying access to effective preventive and treatment therapeutics.

**Methods:**

Using data from the prospective ACORN-HAI cohort across 19 Asian countries between September 2022 and February 2025, we emulated a target trial comparing early guideline-recommended best available therapy (BAT) initiated within 24 h of blood culture collection (early BAT), versus no early BAT among adults with monomicrobial BSIs due to third-generation cephalosporin-resistant *Enterobacterales* (3GCRE), carbapenem-resistant *Enterobacterales* (CRE), or carbapenem-resistant *Acinetobacter* spp. (CRA). Confounding was addressed using inverse probability weighting. The primary outcome was 14-day all-cause mortality. Whether mortality benefit differed according to disease severity was assessed using relative excess risk due to interaction (RERI).

**Findings:**

Among 1412 adults (706 3GCRE, 442 CRE, and 264 CRA BSIs), early BAT was associated with lower 14-day mortality (18.5% versus 25.6% without early BAT; risk difference [RD] −7.1%; 95% CI −11.3%, −3.0%). In 3GCRE BSIs, the mortality reduction was 7.4% (RD −7.4%; 95% CI −12.2, −2.5). Among carbapenem-resistant Gram-negative bacteria (CR-GNB) BSIs, where early BAT predominantly consisted of polymyxin-based regimens (81.7%, 147/180), the estimated RDs were −3.3% (95% CI −13.8, 10.4) for CRE and −7.5% (95% CI −19.1, 4.0) for CRA. Interaction analyses showed a greater mortality benefit of early BAT in severe 3GCRE infections (RERI 2.06, 95% CI 0.47, 4.41), but not in CR-GNB BSIs.

**Interpretation:**

Early BAT was associated with reduced mortality in MDR-GNB BSIs overall. By pathogen group, a mortality reduction was observed in 3GCRE BSIs, whereas findings for CR-GNB BSIs were uncertain. In the context of predominantly polymyxin-based therapy, reducing treatment delays alone may therefore be insufficient to improve outcomes in CR-GNB BSIs, highlighting the need to develop and optimise prevention and therapeutic strategies, especially across AMR-prevalent settings.

**Funding:**

10.13039/100010269Wellcome Trust, 10.13039/501100001352National University of Singapore, Yong Loo Lin School of Medicine, 10.13039/100016017Duke-NUS Medical School, Nanyang Technological University (Lee Kong Chian School of Medicine), Singapore National Centre for Infectious Diseases, and Singapore Medical Research Council.

**Research in context**

**Evidence before this study**

We searched PubMed from database inception to May 30, 2026, using combinations of the terms “bloodstream infection”, “bacteraemia”, “Gram-negative”, “multidrug-resistant”, “appropriate antibiotic therapy”, “delayed therapy”, and “time to appropriate therapy”. We also reviewed international sepsis and multidrug-resistant Gram-negative bacterial (MDR-GNB) infection guidelines.

We identified 73 retrospective studies, nine prospective studies, and five systematic reviews. Findings were highly heterogenous. The largest retrospective study, conducted in the USA and including 14,030 patients with Gram-negative BSIs, reported lower in-hospital mortality with appropriate empirical therapy (adjusted OR 0.52 [95% CI 0.42, 0.64]), with benefit in ceftriaxone-resistant Gram-negative infections but not carbapenem-resistant Enterobacterales (CRE) or *Acinetobacter* spp. (CRA). Among prospective studies, six of nine reported increased mortality associated with delayed or inappropriate antibiotic therapy, and three found no association. We identified only two target trial emulations addressing survival bias. One included 1203 predominantly multidrug-resistant *Acinetobacter* spp. BSIs from a single centre in Thailand, and found a 6.6% (95% CI 0.2, 13.0) increase in 30-day mortality with delays in concordant therapy. The other included 680 Gram-negative bacterial BSIs (50.1% carbapenem-resistant Gram-negative bacteria [CR-GNB]) from 22 Turkish centres, and reported lower mortality with early appropriate therapy but no survival benefit with polymyxin-based regimens.

Current sepsis guidelines recommend prompt appropriate antimicrobial therapy for septic shock and probable sepsis, whereas recommendations for empirical MDR Gram-negative coverage are “conditional” and supported by “very low-certainty evidence”. Treatment guidelines for MDR Gram-negative infections consider polymyxin-based regimens as last-line therapy for CR-GNB infections when newer agents are unavailable, based on low-certainty evidence.

**Added value of this study**

We emulated a target trial to determine whether administering best available therapy (BAT) within 24 h of blood culture collection improves mortality in MDR-GNB BSIs. BAT was defined as receipt of guideline-recommended therapy. Among 1412 adults enrolled from 40 hospitals in 19 countries with monomicrobial BSIs caused by third-generation cephalosporin-resistant Enterobacterales (3GCRE), CRE, or CRA, early BAT was associated with lower mortality overall (risk difference [RD] −7.1%, 95% CI −11.3, −3.0). The benefit of early BAT amongst these MDR-GNB BSIs was observed in 3GCRE BSIs (RD −7.4%, 95% CI −12.2, −2.5), while effects were less conclusive in CRE (RD −3.3%, 95% CI −13.8, 10.4) and CRA BSIs (RD −7.5%, 95% CI −19.1, 4.0). In these carbapenem-resistant BSIs, BAT consisted predominantly of polymyxin-based regimens (81.7%, 147/180). The mortality benefit of early BAT was greater in patients with more severe 3GCRE infections (RERI 2.06, 95% CI 0.47, 4.41), but this benefit was not observed in carbapenem-resistant BSIs.

**Implications of all the available evidence**

In the absence of ethically feasible randomised controlled trials comparing early versus delayed appropriate antibiotic therapy, our target trial emulation provides robust evidence to support earlier microbiological diagnosis especially when effective antibiotic therapies are available, such as for patients with 3GCRE BSIs and higher disease severity. However, the uncertainty in the estimated mortality benefit for CR-GNB BSIs highlights important limitations of current therapeutic options, particularly in regions with high prevalence of AMR. These findings imply that earlier diagnosis alone may be insufficient, and highlight the urgent need to develop and evaluate more effective preventive and therapeutic strategies to establish evidence-based standards of care for CR-GNB infections.

## Introduction

Bloodstream infections (BSIs) caused by multidrug-resistant Gram-negative bacteria (MDR-GNB) are associated with substantial morbidity and mortality. Among MDR-GNB, third-generation cephalosporin-resistant Enterobacterales (3GCRE), carbapenem-resistant Enterobacterales (CRE), and carbapenem-resistant *Acinetobacter baumannii* (CRAB) account for a major proportion of the global antimicrobial resistance (AMR) burden, particularly in low- and middle-income countries (LMICs).^1^, ^2^ Owing to their clinical impact and limited treatment options, these organisms have been designated as critical-priority pathogens by the World Health Organization.^3^

Timely administration of appropriate antibiotic therapy is widely considered a key determinant of outcomes in severe bacterial infections.^4^ Several observational studies have reported higher mortality associated with delays in appropriate antibiotic therapy among patients with BSIs, particularly those who are critically ill.^5^, ^6^, ^7^ However, achieving timely appropriate therapy in MDR-GNB infections remains challenging because resistance is often not recognised until microbiological results become available. Furthermore, the evidence supporting the clinical benefits of earlier appropriate therapy in this setting remains limited. Treatment decisions must therefore balance the potential benefits of prompt effective antibiotic therapy against the risks of toxicity and selection for AMR.^8^

Rapid diagnosis and the availability of effective antimicrobial treatment are fundamental components of timely appropriate therapy. However, microbiological pathogen identification and antimicrobial susceptibility testing (AST) are typically available only several days after culture collection.^9^ In many LMICs, limited microbiology laboratory capacity further delays or compromises the accuracy of AST results, resulting in frequent broad-spectrum antibiotic use without microbiological confirmation of susceptibility.^10^, ^11^ Even when resistance is identified promptly, effective treatment options may be limited. The widespread emergence of carbapenem resistance, especially among *A. baumannii* and Enterobacterales, has substantially reduced the availability of reliable standard-of-care regimens,^12^ prompting the development of novel agents such as β-lactam/β-lactamase inhibitor combinations. However, access to these agents remains limited in many settings, and older drugs such as polymyxins, continue to be widely used for the treatment of CRE and carbapenem-resistant *Acinetobacter* spp. (CRA) infections despite their well-recognised toxicity.^13^

Clarifying the clinical impact of early effective therapy in MDR-GNB BSIs is therefore essential.

Evidence of benefit associated with early treatment could help define the role of and investments in rapid diagnostic technologies, which can shorten time to effective therapy, but whose impact on survival remains uncertain.^14^ Conversely, limited benefit despite early therapy with the best currently available antibiotics would suggest that optimising overall patient management should be prioritised beyond reducing treatment delays alone, including accelerating the development and evaluation of more effective preventive and antimicrobial therapies and establishing evidence-based standards of care.

Randomised controlled trials directly comparing early versus delayed appropriate therapy are not ethically feasible because treatment allocation cannot deliberately delay appropriate antimicrobial treatments. Causal inference approaches, such as target trial emulation (TTE), provide a framework to address this challenge by emulating the design and analysis of a randomised trial using observational data.^15^ TTE is particularly suited to evaluate timing and choice of antimicrobial therapy because these treatment decisions are influenced by available clinical and microbiological information. Early broad-spectrum therapy may be more likely in patients with greater illness severity or at higher risk for MDR infection, factors that are also associated with patient outcomes, leading to confounding by indication and potentially biased observational estimates.

In this study, we investigated whether initiation of guideline-recommended best available therapy (BAT) within 24 h of blood culture collection reduces short-term mortality among hospitalised adults with MDR-GNB bloodstream infections. To address this question, we emulated a target trial among patients with bloodstream infections caused by 3GCRE, CRE, or CRA enrolled across a large multinational network.

## Methods

### Study setting

We used data from ACORN-HAI (A Clinically-Oriented Antimicrobial Resistance Surveillance Network for Healthcare-Associated Infections), a multicentre cohort study involving 41 hospitals across 19 Asian countries.^16^ Between September 2022 and February 2025, the study prospectively enrolled 10,111 patients with healthcare-associated or hospital-acquired BSIs, or ventilator-associated pneumonia, collecting clinical, microbiological, antibiotic treatment, and outcome data. The overall study design of ACORN-HAI has been described previously.^17^ Ethics approval was obtained from the Singapore National Healthcare Group Domain Specific Review Board (2022/00276), Oxford University Oxford Tropical Research Ethics Committee (527-22), and local ethics committee at each participating site. A waiver of informed consent was sought from each hospital's ethics committee because the study was observational and involved minimal-risk use of routinely collected clinical data, with approvals obtained according to local regulations. The study was conducted in accordance with the principles of the Declaration of Helsinki and the Good Clinical Practice guidelines.

Details on participating hospitals are shown in Supplementary Table S1.

### Study design

We conducted a target trial emulation to estimate the causal effect of early BAT on mortality. A hypothetical target trial was specified according to the framework proposed by Hernán and Robins and emulated using observational data from the ACORN-HAI cohort.^14^ The protocol of the target trial and its emulation are summarised in Supplementary Table S2. Reporting followed the TARGET Statement for transparent reporting of observational studies emulating a target trial.^18^

### Eligibility criteria and time zero

Eligible participants were adult patients (≥18 years) with hospital-acquired or healthcare-associated monomicrobial BSI caused by 3GCRE, CRE, or CRA. Hospital-acquired BSI was defined as blood cultures collected more than two days after admission, while healthcare-associated BSI referred to blood cultures collected within two days of admission in patients with recent healthcare exposure. The latter included, within the three months before admission, regular healthcare contact (e.g., dialysis, outpatient antibiotic therapy, or chemotherapy), admission to another healthcare facility, or surgical procedures.^17^ Resistance phenotypes were defined using phenotypic resistance criteria from the Clinical and Laboratory Standards Institute (CLSI) or European Committee on Antimicrobial Susceptibility Testing (EUCAST) according to local conventions (Supplementary Table S3). Only the first BSI episode per patient during the study period was included.

Time zero (Day 0) was defined as infection onset, which was the sample collection date of the first blood culture that subsequently yielded one of the study pathogens. Although clinical infection onset might have preceded blood culture collection, Day 0 was defined using blood culture collection date as a standardised proxy for infection onset in our target trial emulation framework because the onset of clinical signs and symptoms is heterogeneous and difficult to ascertain reliably from routinely collected data. Patients who died or were discharged within one calendar day of Day 0 were excluded, as they could not plausibly initiate BAT within the exposure window.

### Treatment strategies

The target trial compared two treatment strategies: initiation of BAT on Day 0 versus non-initiation of BAT on that day. Consistent with previous studies evaluating timing of appropriate therapy in BSIs,^19^, ^20^ treatment timing was evaluated using a 1-day window, acknowledging that clinical recognition of sepsis and blood culture collection may not occur simultaneously. Hence in the primary analysis, a ±1-day grace period around Day 0 was allowed to account for operational delays in blood culture collection and antibiotic administration.

BAT was defined as initiation of the guideline-recommended antibiotic treatment expected to be active against the identified pathogen and resistance phenotype (Table 1). This pragmatic, real-world approach was adopted because reliable *in vitro* susceptibility testing for polymyxins and β-lactam/β-lactamase inhibitor combinations against MDR pathogens was frequently unavailable across the participating sites, particularly in LMICs, making susceptibility-based definitions prone to differential exposure misclassification. To assess the robustness of this approach, we performed a sensitivity analysis defining BAT according to available *in vitro* susceptibility results.

**Table 1:** Definition of best available antibiotic therapy across pathogen groups.

Pathogen group	Best available antibiotic therapy definition
Carbapenem-resistant *Acinetobacter* spp.	Administration of at least one of the following antibiotics: colistin, polymyxin B, ampicillin-sulbactam, or cefoperazone-sulbactam.
Carbapenem-resistant Enterobacterales	Administration of at least one of the following antibiotics: colistin, polymyxin B, or ceftazidime-avibactam.
Third-generation cephalosporin-resistant Enterobacterales	Administration of at least one carbapenem (meropenem, imipenem, or ertapenem).

### Assignment procedures and follow-up

Because treatment strategies were not randomly assigned in this observational cohort, inverse probability of treatment weighting (IPTW) was used to create a pseudo-population in which measured baseline confounders were balanced between treatment strategies, thereby approximating exchangeability. Follow-up started at treatment assignment and continued until day 28 after infection onset. In our dataset, follow-up till day 28 was complete for all included patients.^16^

### Outcomes

The primary outcome was all-cause mortality at 14 days after infection onset. Secondary outcomes included mortality at 7 and 28 days.

### Statistical analysis

The primary causal estimand of interest was the intention-to-treat-like effect of initiating versus not initiating BAT on the day of blood culture collection, expressed as the absolute risk difference in all-cause mortality at 7, 14, and 28 days. Accordingly, patients were analysed according to their treatment strategy at baseline irrespective of subsequent changes in antibiotic therapy.

Baseline confounding was addressed by adjusting for prespecified covariates selected using a directed acyclic graph and measured at or before Day 0, including demographics, comorbidity burden, acute illness severity (Pitt Bacteraemia Score, quick Sequential Organ Failure Assessment [qSOFA], intensive care unit [ICU] admission, and mechanical ventilation at infection onset), prior inpatient antibiotic exposure (number of days of antibiotic therapy from hospital admission to BSI onset), length of stay before BSI onset and admission characteristics (Supplementary Fig. S1). Before weighting, propensity score overlap between treatment strategies was assessed by visual inspection of the propensity score distributions. After weighting, covariate balance was assessed and considered adequate with standardised mean differences below 0.1 for all prespecified baseline variables (Supplementary Fig. S2).

IPTW based on the propensity score was used to emulate random assignment of treatment strategies at baseline. Propensity scores were estimated using logistic regression including all prespecified baseline covariates. Stabilised weights were calculated as the marginal probability of treatment divided by the conditional probability of treatment given baseline covariates. Continuous covariates were assessed prior to weight estimation with respect to their distribution and functional form. Length of stay before BSI onset was log-transformed due to its markedly skewed distribution, while prior antibiotic exposure was modelled using cubic splines with three degrees of freedom to allow for non-linearity. Collinearity was assessed using generalised variance inflation factors adjusted for degrees of freedom; all values were below the threshold of 2 (maximum 1.47), indicating low collinearity among covariates. In the primary analysis, stabilised weights were truncated at the 1st and 99th percentiles to reduce the influence of extreme weights. Expected mortality risks under each treatment strategy were estimated as IPT-weighted marginal risks. Absolute risk differences were calculated by comparing the weighted risks, with 95% confidence intervals obtained by non-parametric bootstrap resampling (1000 replicates).

Analyses were performed both in the overall cohort, combining all pathogen groups, and separately for BSIs caused by 3GCRE, CRE, and CRA. The pooled analysis estimated the population-average effect of early BAT across all included MDR-GNB BSIs, reflecting the clinical setting in which pathogen identification and susceptibility were unavailable at treatment initiation. Pathogen-stratified analyses were undertaken because the antibiotic therapies constituting BAT, baseline mortality risks, and clinical characteristics were expected to differ substantially across pathogen groups. For effect-modification analyses within pathogen groups, weighted log-binomial regression models including treatment strategy, qSOFA (≥2 versus <2), and their interaction term were fitted (using stabilised weights with the numerator conditioned on qSOFA), and RERIs and 95% confidence intervals were estimated using the method of variance estimates recovery.^21^, ^22^ Given the limited availability of several laboratory variables required to calculate SOFA across participating sites, qSOFA was used as a pragmatic measure for acute illness severity, as it relies on bedside clinical parameters and has been previously evaluated across resource-limited settings.^23^ An additional interaction analysis using Pitt bacteraemia score (≥3 versus <3) as an alternative measure of baseline severity was performed.

Sensitivity analyses assessed the robustness of the results to alternative exposure definitions and assumptions, including restricting the treatment assignment window to initiation exclusively on Day 0 (1) or exclusively on Day 1 (2) without a grace period; requiring received agents included in the BAT definition to be concordant with available *in vitro* susceptibility results (intermediate or susceptible) (3); defining antibiotic appropriateness based on available *in vitro* susceptibility results (intermediate or susceptible) among clinically plausible antibiotics, irrespective of the guideline-based BAT definition (Supplementary Table S4) (4); defining early BAT as initiation on Day 0 (±1 day) with continuation for at least 3 consecutive days among patients who remained alive and hospitalised through the first 96 h after infection onset (5), and repeating the primary analyses using untruncated stabilised weights (6). Because there were no missing mortality outcome data and missingness in baseline covariates was low (3.0%), with missing data limited to Pitt Bacteraemia Score and admission type, we performed complete-case analysis.

Analyses were performed using R software (R Core Team, 2025; version 4.5.1). All analysis codes are publicly available on GitHub (https://github.com/advanceid).

### Role of the funding source

The funders had no role in study design, data collection, data analysis, data interpretation, writing of the report, or the decision to submit the manuscript for publication.

## Results

Of the 10,111 patients enrolled in the prospective cohort, 1412 patients from 40 sites across 19 countries fulfilled the eligibility criteria and were included in the analysis (Fig. 1). Of these BSI episodes, 706/1412 (50.0%) BSIs were caused by 3GCRE, 442/1412 (31.3%) by CRE, and 264/1412 (18.7%) by CRA. Most patients were recruited from upper- and lower-middle-income countries (735/1412 [52.1%] and 550/1412 [39.0%], respectively). Overall, 558/1412 (39.5%) patients were admitted to the ICU at infection onset, with the highest proportion among patients with CRA BSIs (64.0%, 169/264), followed by CRE BSIs (38.9%, 172/442) and 3GCRE BSIs (30.7%, 217/706) (Table 2, Supplementary Table S5). Median length of stay before infection onset was 8 days (interquartile range [IQR] 2–16) overall and was shorter amongst those with 3GCRE BSI (5 days, IQR 0–14) than carbapenem-resistant Gram-negative bacteria (CR-GNB) BSI (11 days, IQR 4–19). Compared with patients with CRE and 3GCRE BSIs, those with CRA BSIs had higher median Pitt bacteraemia (2 [IQR 0–6] versus 1 [0–3] and 1 [0–2], respectively) and quick SOFA (2 [1–2] versus 1 [0–2] for both CRE and 3GCRE BSIs) scores at infection onset. The median time from blood culture collection to availability of AST results was 3 days across all pathogens (IQR 2–3 for 3GCRE BSIs; IQR 2–4 for both CRE and CRA BSIs).

**Fig. 1:**
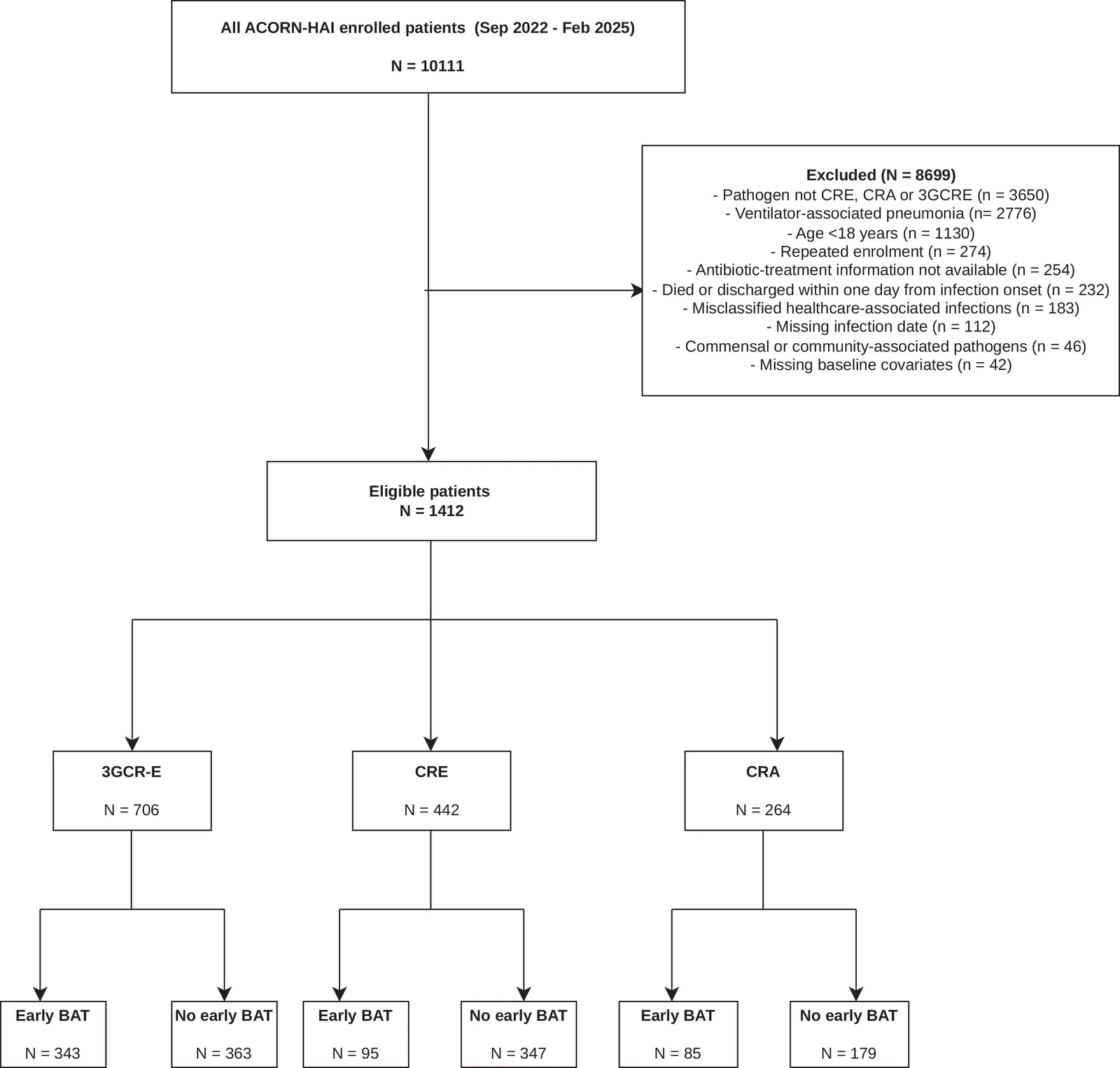
Study flow diagram. **Abbreviations:** CRE, carbapenem-resistant Enterobacterales; CRA, carbapenem-resistant *Acinetobacter* spp.; 3GCRE, third-generation cephalosporin resistant Enterobacterales; BAT, Best Available Therapy.

**Table 2:** Baseline patient characteristics.

Characteristic	Overall	3GCRE	CRA	CRE
	N = 1412	N = 706	N = 264	N = 442
Age (years), mean (SD)	59.2 (16.6)	61.0 (15.7)	58.4 (17.1)	56.7 (17.3)
Male sex, n (%)	786 (56%)	358 (51%)	158 (60%)	270 (61%)
Country income level, n (%)				
High income	127 (9%)	98 (14%)	9 (3%)	20 (5%)
Lower middle income	550 (39%)	224 (32%)	103 (39%)	223 (50%)
Upper middle income	735 (52%)	384 (54%)	152 (58%)	199 (45%)
Charlson Comorbidity Index, median (IQR)	1 (0–2)	1 (0–2)	1 (0–2)	1 (0–2)
Quick SOFA, median (IQR)	1 (0–2)	1 (0–2)	2 (1–2)	1 (0–2)
Pitt Bacteraemia Score, median (IQR)	1 (0–3)	1 (0–2)	2 (0–6)	1 (0–3)
Enterobacterales species, n (%)				
*E.coli*	_	410 (58%)	_	129 (29%)
*Klebsiella* spp.	_	233 (33%)	_	265 (60%)
*Enterobacter* spp.	_	29 (4%)	_	28 (6%)
Other	_	34 (5%)	_	20 (5%)
Length of stay before infection onset (days), median (IQR)	8 (2–16)	5 (0–14)	10 (6–18)	11 (4–19)
Admission ward type, n (%)				
General ward	911 (65%)	537 (76%)	104 (39%)	270 (61%)
ICU	501 (35%)	169 (24%)	160 (61%)	172 (39%)
ICU at infection onset, n (%)	558 (40%)	217 (31%)	169 (64%)	172 (39%)
Start of BAT within 24 h from infection onset, n (%)	523 (37%)	343 (49%)	85 (32%)	95 (21%)

Abbreviations: 3GCRE, Third-generation-cephalosporin-resistant *Enterobacterales*; CRE, Carbapenem-resistant *Enterobacterales*; CRA, Carbapenem-resistant *Acinetobacter spp.*; SD, standard deviation; IQR, Interquartile range; ICU, Intensive Care Unit; BAT, Best Available Therapy; SOFA, Sequential Organ Failure Assessment.

Other Enterobacterales included *Citrobacter* spp. (3GCRE, n = 8; CRE, n = 4), *Edwardsiella* spp. (3GCRE, n = 1; CRE, n = 0), *Morganella* spp. (3GCRE, n = 2; CRE, n = 3), *Pantoea* spp. (3GCRE, n = 1; CRE, n = 0), *Proteus* spp. (3GCRE, n = 16; CRE, n = 5), *Providencia* spp. (3GCRE, n = 1; CRE, n = 0), *Raoultella* spp. (3GCRE, n = 0; CRE, n = 3), and *Serratia* spp. (3GCRE, n = 5; CRE, n = 5).

The types of antibiotic therapy at infection onset differed between patients with 3GCRE bloodstream infections and those with CR-GNB (Fig. 2). Early BAT was administered in 48.6% (343/706) of patients with 3GCRE BSIs compared with 25.5% (180/706) of those with CR-GNB BSIs. Among patients with 3GCRE BSIs receiving carbapenem-based early BAT, 178/343 (51.9%) received combination therapy with at least one additional anti-Gram-negative agent, while 165/343 (48.1%) received carbapenem-monotherapy (Supplementary Table S6). Among CR-GNB BSIs, use of international guideline recommended agents, such as ceftazidime-avibactam for CRE or sulbactam-based regimens for CRA, was infrequent (3.6% [16/442] and 6.4% [17/264], respectively). Cefiderocol, sulbactam-durlobactam and aztreonam-avibactam were not used at any study site during the study period.

**Fig. 2:**
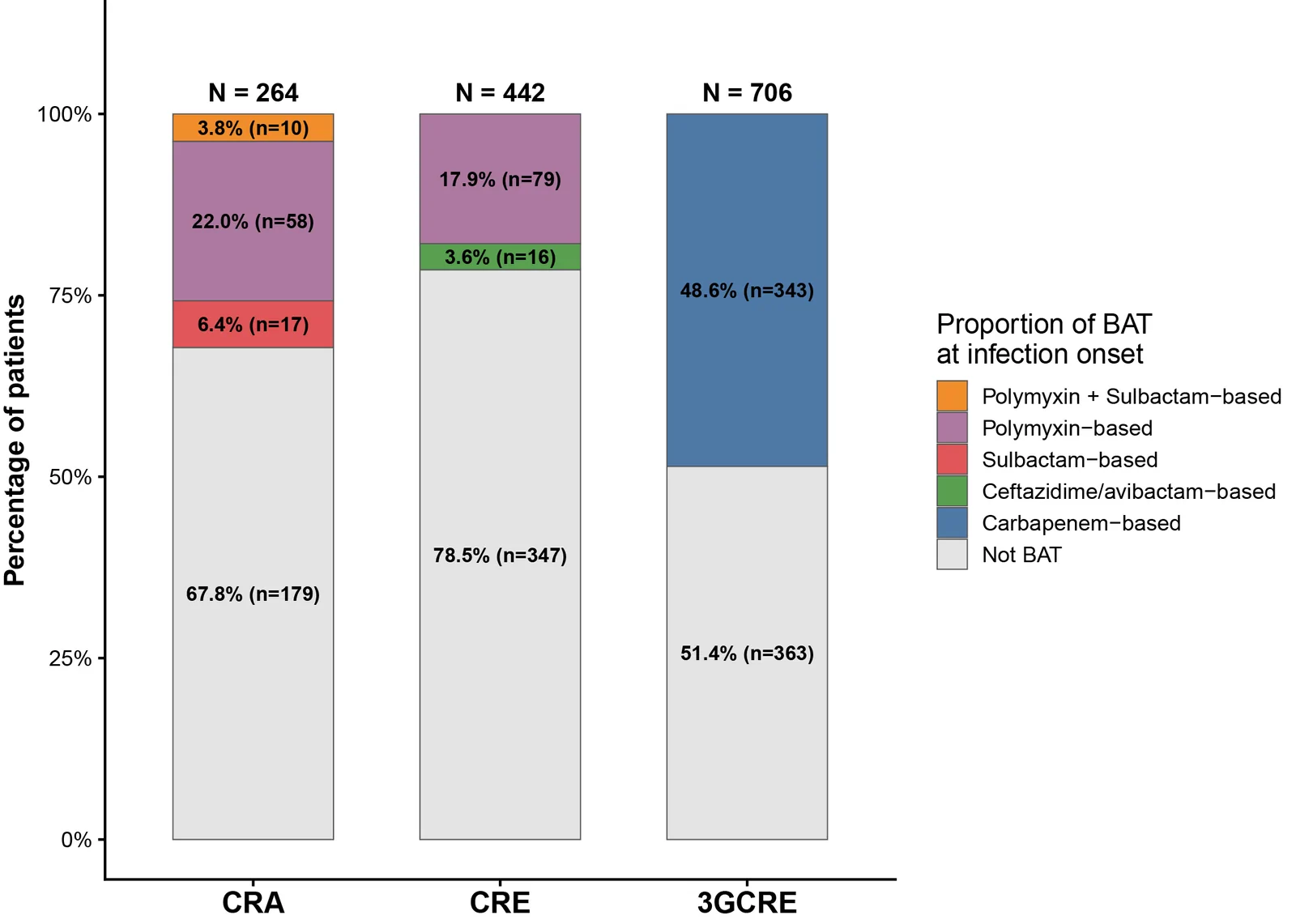
Proportion of best available therapy at infection onset. Polymyxin-based therapy included colistin (polymyxin E) and polymyxin B. **Abbreviations:** BAT, best available therapy; CRE, carbapenem-resistant Enterobacterales; CRA, carbapenem-resistant Acinetobacter spp.; 3GCRE, third-generation cephalosporin resistant Enterobacterales.

Overall, patients receiving early BAT had a median of 14 days (IQR 8–20) of total antibacterial therapy, compared with 11 days (IQR 7–17) among those not receiving early BAT (Supplementary Table S7). Days of therapy for the different BAT agents through Day 14, stratified by pathogen group, are shown in Supplementary Fig. S3.

Among patients who did not receive BAT at infection onset, carbapenem-based regimens accounted for 60.3% (108/179) of CRA BSIs and 57.9% (201/347) of CRE BSIs, while piperacillin-tazobactam-based regimens accounted for 52.9% (192/363) of 3GCRE BSIs.

In the unadjusted analysis, there were no major differences in the crude 14-day mortality between patients who received early BAT and those who did not in the overall cohort (Supplementary Fig. 4A). Amongst the pathogen groups, patients with CRE BSIs had higher 14-day mortality among those who received early BAT compared to those without at infection onset (Supplementary Fig. 4B, Supplementary Table S8).

The target trial emulation showed that early BAT was associated with a consistently lower mortality at the various follow-up time points (Fig. 3). At Day 14, the estimated mortality risk was 18.5% (95% CI 15.4%, 21.6%) with early BAT and 25.6% (95% CI 22.8%, 28.7%) without early BAT, corresponding to an absolute mortality reduction of 7.1% (95% CI 3.0%, 11.3%). Similar findings were observed at Day 7 (mortality 11.1%, 95% CI 8.6%, 13.7% versus 16.4%, 95% CI 13.9%, 19.1%, respectively; mortality reduction 5.3%, 95% CI 1.6%, 8.8%) and Day 28 (mortality 27.4%, 95% CI 23.7%, 31.1% versus 33.4%, 95% CI 30.1%, 36.8%, respectively; mortality reduction 5.9%, 95% CI 1.1%, 11.3%).

**Fig. 3:**
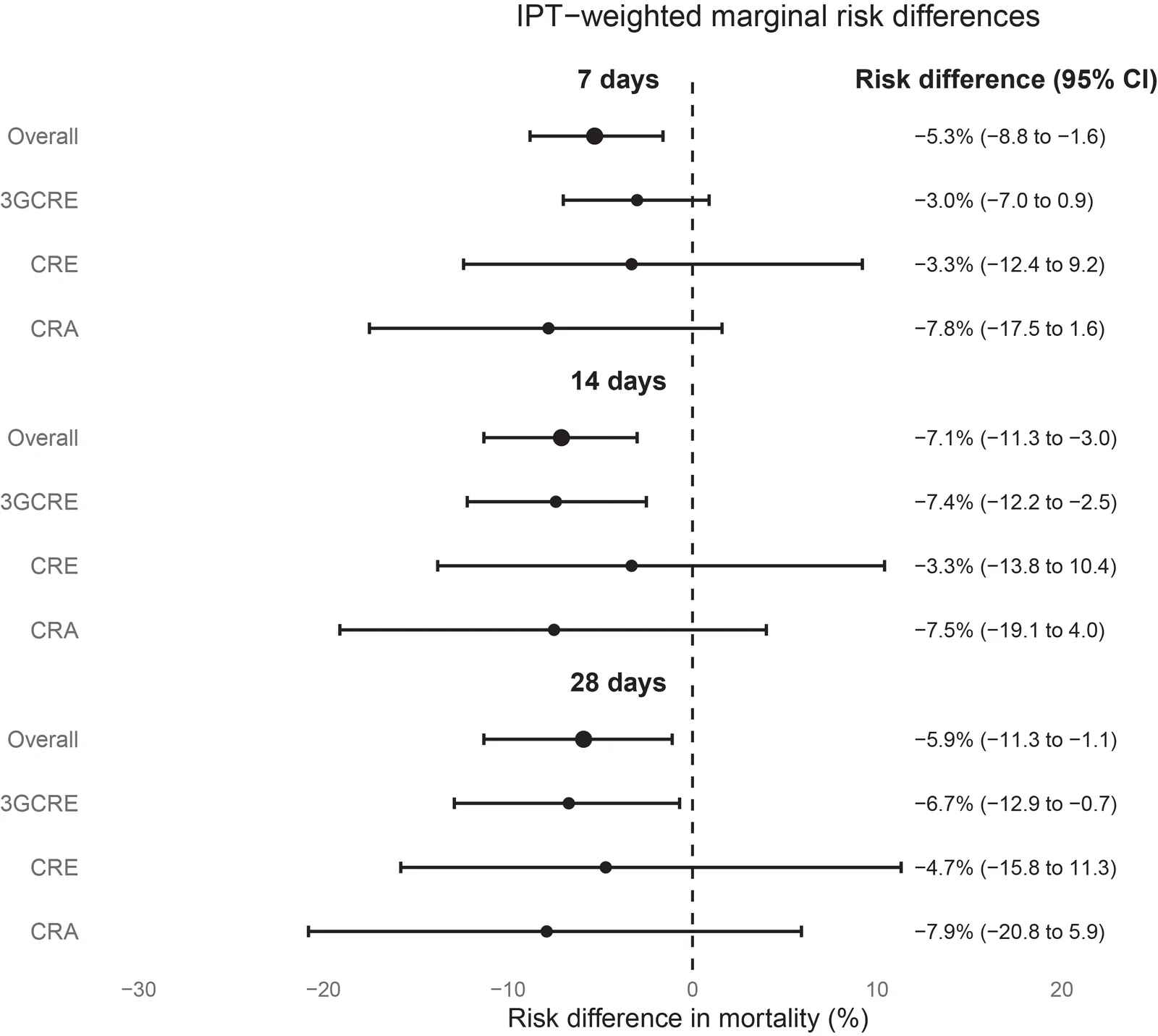
IPT-weighted marginal risk differences for 7-, 14-, and 28-day mortality overall and by pathogen group. **Abbreviations:** BAT, best available therapy; CRE, carbapenem-resistant Enterobacterales; CRA, carbapenem-resistant Acinetobacter spp.; 3GCRE, third-generation cephalosporin-resistant Enterobacterales; RD, risk difference; CI, confidence interval.

Stratified by pathogen group, early BAT was associated with an absolute mortality reduction of 7.4% among patients with 3GCRE BSIs (mortality 9.9%, 95% CI 6.9%, 13.3% versus 17.3%, 95% CI 13.5%–21.4% without early BAT; mortality reduction 7.4%, 95% CI 2.5%, 12.2%) at Day 14. In contrast, estimates among patients with CR-GNB BSIs were uncertain, with RDs of −3.3% (95% CI −13.8%, 10.4%) for CRE and −7.5% (95% CI −19.1%, 4.0%) for CRA BSIs. In interaction analyses, the absolute mortality benefit of early BAT was greater among patients with 3GCRE BSIs and high baseline qSOFA than among those with low baseline qSOFA (RERI 2.06, 95% CI 0.47, 4.41; Fig. 4A; Supplementary Table S9). There was no additive interaction among patients with CRE BSIs (RERI −0.92, 95% CI −4.40, 0.21; Fig. 4B; Supplementary Table S10) or CRA BSIs (RERI −0.06, 95% CI −3.50, 1.58; Fig. 4C; Supplementary Table S11) in terms of disease severity. Findings were consistent in additional analyses using the Pitt bacteraemia score as an alternative measure of baseline severity (Supplementary Tables S12–S14).

**Fig. 4:**
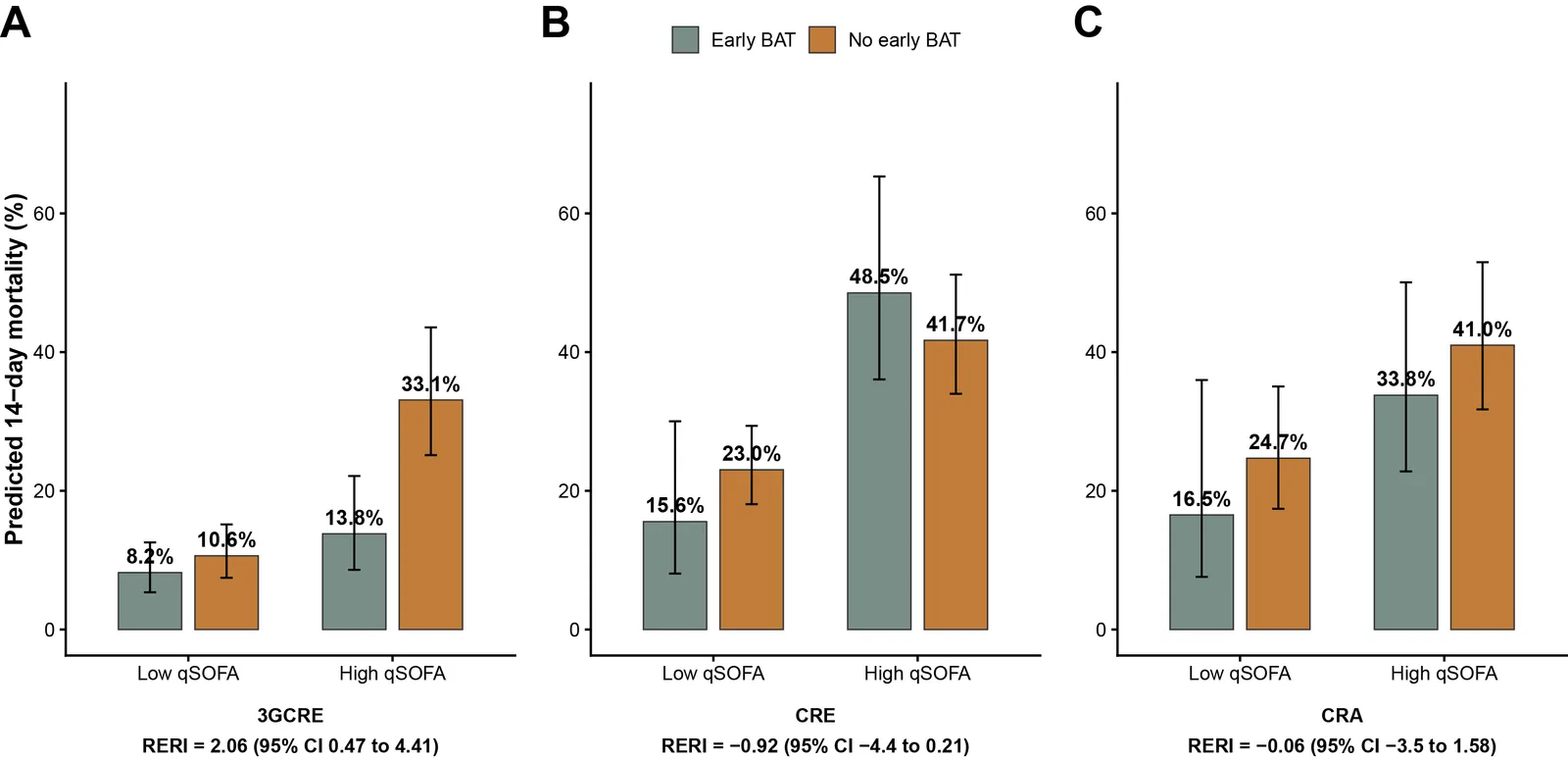
Predicted 14-day mortality from IPT-weighted regression models according to receipt of early best available therapy and baseline severity (qSOFA <2 versus ≥2), stratified by pathogen group: (A) third-generation cephalosporin-resistant Enterobacterales (3GCRE), (B) carbapenem-resistant Enterobacterales (CRE), and (C) carbapenem-resistant Acinetobacter spp. (CRA). The relative excess risk due to interaction (RERI) is shown for each pathogen group. **Abbreviations:** BAT, best available therapy; 3GCRE, third-generation cephalosporin-resistant Enterobacterales; CRE, carbapenem-resistant Enterobacterales; CRA, carbapenem-resistant Acinetobacter spp.; qSOFA, quick Sequential Organ Failure Assessment; BSIs: bloodstream infections; RERI: relative excess risk due to interaction; CI: confidence interval.

The overall mortality benefit associated with early BAT was robust in all six sensitivity analyses (Supplementary Tables S15–S20). Across alternative exposure definitions, including treatment initiation on Day 0 only, Day 1 only, or according to *in vitro* susceptibility, and the per-protocol-like analysis, early BAT was consistently associated with an absolute reduction in 14-day mortality of approximately 6–8% in the overall cohort. Similar patterns were observed within pathogen groups, with mortality estimates generally lower with early BAT, although effects for CRE and CRA BSIs were imprecise, with wide confidence intervals compatible with both benefit and harm.

## Discussion

In this large target trial emulation using data from 1412 patients with MDR-GNB BSI enrolled in a multinational prospective cohort, BAT on the day of blood culture collection was associated with lower mortality compared with no early BAT. This mortality reduction was mainly contributed by patients with 3GCRE BSIs. In effect modification analyses, a greater mortality benefit of early BAT with increasing disease severity was observed only amongst patients with 3GCRE BSIs.

Several explanations may underlie the observed differences between patients with 3GCRE and CR-GNB BSIs. First, patients with CR-GNB BSIs represented a clinically distinct population, with longer hospitalisation before infection onset, greater illness severity, and more frequent ICU admission than those with 3GCRE BSIs. These differences suggested that mortality in CR-GNB infections may be influenced by factors beyond timely antimicrobial therapy alone. Second, the therapies constituting BAT differed substantially between pathogen groups. Among patients with 3GCRE BSIs, early BAT primarily consisted of carbapenems, which were widely available and recommended as first-line therapy.^24^, ^25^ In contrast, BAT for CR-GNB BSIs largely comprised older agents such as polymyxins, which are associated with substantial toxicity and are no longer recommended when newer alternatives are available.^24^, ^25^ Thus, the observed estimates in CR-GNB BSIs may partly reflect the limited clinical effectiveness of these regimens rather than the effect of treatment timing alone. Thirdly, although residual confounding cannot be excluded, the target trial emulation framework was designed to minimise biases common to conventional observational analyses, including immortal time bias and confounding by indication. The higher unadjusted mortality observed with early BAT among patients with CRE BSIs may partly reflect such confounding, as patients with greater illness severity were more likely to receive early broad-spectrum antibiotics. After adjustment using IPTW, the effect of early BAT remained uncertain among patients with CR-GNB BSIs; point estimates suggested lower mortality, but confidence intervals were compatible with both benefit and harm. Nevertheless, residual confounding cannot be excluded, as the available measures may not have fully captured differences in acute illness severity between treatment groups, and the direction of any resulting bias cannot therefore be reliably predicted.

Our findings are broadly consistent with previous studies demonstrating that delayed appropriate antibiotic therapy is associated with increased mortality in GNB BSI.^5^, ^6^, ^7^, ^26^ However, evidence in MDR-GNB BSIs remains inconsistent across studies with varying designs, treatment definitions, and treatment timing. Two previous observational studies evaluated treatment delays in this setting using target trial emulation designs similar to ours.^10^, ^27^ In a Thai cohort of 1203 patients with hospital-acquired *Acinetobacter* bacteraemia, mostly carbapenem-resistant, delayed in-vitro concordant therapy was associated with a 6.6% (95% CI 0.2–13.0) increase in 30-day mortality.^10^ This effect was similar in magnitude to our estimate for CRA BSIs, although our smaller CRA sample yielded a more uncertain estimate. However, the antibiotics constituting concordant therapy among patients with carbapenem-resistant isolates were not detailed, limiting direct comparison according to treatment regimen. A multicentre study from Türkiye similarly reported lower mortality with early appropriate therapy among patients with GNB BSIs, including in analyses of CR-GNB.^27^ Interestingly, when the analysis was restricted to polymyxin-based regimens, the estimated benefit was less evident (14-day aHR 0.74, 95% CI 0.44–1.24),^27^ consistent with the uncertainty in our estimates for CR-GNB BSIs, where BAT predominantly consisted of polymyxins. Both previous studies were restricted to hospital-acquired BSIs within individual countries and were conducted in settings with limited access to newer antibiotics, although they also included non-MDR GNBs. Our study extends this evidence specifically to MDR-GNB hospital- or healthcare-associated BSIs across similar settings in 19 countries, including LMICs. Other observational studies in CRE and *Acinetobacter* spp. BSIs, where treatment relied predominantly on older agents such as tigecycline and polymyxins, generally failed to demonstrate a mortality benefit of earlier appropriate therapy.^6^, ^28^ In contrast, a more recent study of BSIs caused by metallo-β-lactamase-producing pathogens, in which patients were treated predominantly with newer antimicrobial agents, found that delayed appropriate therapy was associated with increased mortality.^29^ Recently, a large US cohort of patients with difficult-to-treat (DTR) Gram-negative infections reported persistent delays to appropriate therapy despite increasing availability of newer antibiotics and found no survival benefit associated with concordant initial therapy. The authors attributed this finding, at least in part, to the predominance of traditional DTR-active agents over newer antibiotics among patients receiving concordant therapy (71%), as well as the relatively low prevalence of septic shock.^30^ Our finding that the benefit of early BAT was greater among 3GCRE patients with more severe disease is consistent with this interpretation and with current sepsis guidelines, which recommended considering illness severity when balancing the benefits and risks of empirical MDR coverage.^8^ The different interaction pattern observed in CR-GNB BSIs, however, suggests that the role of disease severity may also depend on resistance and treatment patterns.

Our results have important implications for research and clinical practice. Advances in rapid diagnostic technologies capable of providing same-day pathogen identification and antimicrobial susceptibility results have the potential to transform the management of MDR-GNB BSIs by enabling earlier targeted therapy and reducing unnecessary broad-spectrum antibiotic use.^31^ However, a recent RCT on rapid AST found no improvement in 30-day mortality despite shortening time to effective therapy, with no clear mortality benefit also among patients with CR-GNB BSIs.^14^ Together with our findings, this suggests that the clinical benefits of faster diagnosis may depend on factors beyond treatment timing alone, including the effectiveness of available therapies. For this reason, continued investment is needed to develop and rigorously evaluate therapeutic strategies for CR-GNB infections, alongside improving equitable access to effective antimicrobial agents, particularly in LMICs where major gaps in access to both diagnostics and treatment remain.^32^

Nevertheless, our study has several limitations. Firstly, causal interpretation of the IPTW estimates relies on the assumptions of no unmeasured confounding, consistency, positivity, correct specification of the propensity score model, and absence of relevant measurement error. Despite the use of a target trial emulation framework, residual confounding due to unmeasured factors, including incompletely captured disease severity, cannot be excluded. Data on antibiotic dosages and the timing of source control were not available and therefore could not be accounted for. The antibiotic regimens classified as early BAT were heterogeneous, and the smaller pathogen-specific cohorts provided more limited support for comparisons between treatment strategies. However, assessment of propensity-score overlap and covariate balance was performed, with absolute SMD <0.1 after weighting across all analyses. Furthermore, although data were collected prospectively using standardised definitions and monitoring procedures,^16^ some measurement error in clinical and treatment variables remains possible in a large multicentre study. Secondly, resistance was classified using phenotypic criteria and did not capture differences related to specific resistance mechanisms, such as carbapenemase or extended-spectrum β-lactamase types. In addition, BAT was defined at the calendar-day level (rather than hours) and based on guideline-informed assumptions, which may not fully reflect *in vitro* effectiveness. Some patients classified as receiving early BAT may therefore have received an inactive agent, potentially resulting in exposure misclassification and attenuating the estimated effect, particularly for CRE and CRA; however, findings were consistent in a sensitivity analysis requiring documented AST concordance, although estimates were less precise due to the smaller sample size. Thirdly, although ACORN-HAI includes a diverse range of hospitals across Asia, most patients were enrolled in upper- and lower-middle-income countries, where access to newer agents for CR-GNB infections is more limited than in high-income settings.^13^ Our findings may therefore not be fully generalisable to settings where newer antimicrobial agents are routinely used. In addition, the numbers of patients in the CRE and CRA subgroups were smaller than those in the 3GCRE subgroup, resulting in less precise estimates with wide confidence intervals, such that modest treatment effects cannot be excluded. Finally, our analysis was restricted to patients with the MDR phenotypes of interest. We did not evaluate outcomes among patients who received early broad-spectrum therapy but were ultimately found not to have resistant organisms and therefore could not assess the potential clinical or ecological consequences of inappropriate broad-spectrum use (e.g., routine carbapenem administration). This is particularly relevant to empirical treatment decisions, which are made before the causative pathogen and its resistance profile are known. Expanding empirical BAT to reduce treatment delays could therefore increase unnecessary broad-spectrum antibiotic exposure among patients without MDR-GNB infections, with potential individual and ecological consequences. While our study focused on quantifying the mortality effects of early BAT among patients with MDR-GNB BSIs and identifying those most likely to benefit, it could not capture these broader consequences at the population level. Our results should therefore not be interpreted as supporting broader empirical use of BAT in patients without confirmed MDR-GNB infection. This limitation should be considered when applying our findings in practice and underscores the importance of antimicrobial stewardship.

In conclusion, early BAT was associated with reduced mortality among patients with MDR-GNB BSIs, with the greatest benefit observed in 3GCRE BSIs and higher clinical severity. These findings support prioritising prompt effective therapy for patients at high risk of 3GCRE BSI and presenting with higher clinical severity. The uncertain findings among patients with CR-GNB BSIs, in whom BAT largely relied on polymyxin-based regimens, highlight the need to optimise management strategies beyond reducing treatment delays alone, including through preventive strategies, the development and evaluation of more effective antimicrobial therapies, and equitable access to these therapies. As rapid diagnostic technologies evolve towards same-day pathogen identification and antimicrobial susceptibility testing, future clinical trials may become feasible to evaluate treatment strategies through randomisation at infection onset. Until then, target trial emulation provides a robust framework for addressing this important clinical question.

## Contributors

All authors contributed to data collection or interpretation. LM and YM had full access to the complete dataset for data analysis, verified the underlying data in the study, conducted the statistical analyses, and draughted the initial manuscript. Site investigators had access to data from their own sites; access to data from other sites or the complete dataset required a formal request and approval in accordance with the study's data governance and confidentiality procedures. All authors critically revised the manuscript, vouched for the completeness and accuracy of the data and analyses, and participated in the decision to submit the manuscript for publication. All authors read and approved the final version of the manuscript.

## Data sharing statement

De-identified participant-level data will be made available to qualified researchers upon reasonable request after publication. Requests should be submitted to the study data manager (Jie Su) and will be reviewed by the data governance committee and ADVANCE–ID directors. Data sharing is subject to institutional approvals, data use agreements, and compliance with applicable ethical and regulatory requirements. Analysis code is publicly available via GitHub at (https://github.com/advanceid).

## Declaration of interests

CLA reports honoraria for lectures/speakers bureaus from MSD and Pfizer Philippines and membership of the ADVANCE–ID Strategic Advisory Committee. GSR reports research funding from the National Institutes of Health, United States, and the National Institutes of Health, Malaysia, for *Plasmodium knowlesi* malaria research; speaker activities at the International Congress of Malaria & Tropical Medicine, Asia Dengue Summit 2026, and ESCMID Global 2024; mentor activities at the International Pandemic Science Conference 2026; membership of the ADVANCE–ID Strategic Advisory Committee and ADAPT Trial Steering Committee; and a role as Country Ambassador for the Royal Society of Tropical Medicine and Hygiene, United Kingdom. ALHK reports consulting fees and support for attending meetings and/or travel from Pfizer (paid to institution). YDo reports grants or contracts from Innoviva (paid to institution); consulting fees from GSK, Shionogi, Meiji Seika Pharma, and Roche; and honoraria for lectures, presentations, speakers bureaus, manuscript writing, or educational events from Shionogi, Gilead, BD, and Chugai. HH reports consulting fees from Solventum and honoraria for lectures, presentations, speakers bureaus, manuscript writing, or educational events from Solventum, Moderna, and Shionogi. DLP reports institutional grants from Pfizer, Shionogi, Merck, Gilead, BioVersys, and bioMerieux; consulting fees from AMR Action Fund, GARDP, CARB-X, Basilea, Aurobac, Antabio, BioVersys, Gangagen, Menarini, ADVANZ, and Tamrisa; honoraria from Pfizer, Shionogi, and Merck; travel support from GSK, Shionogi, and Pfizer; and an honorary leadership role in the Australasian Society for Infectious Diseases. All other authors declare no competing interests.

## Appendix A Supplementary data

Supplementary

Group Authorship
