# Supplementary material for: Early best available antibiotic therapy for multidrug-resistant gram-negative bloodstream infections: a target trial emulation
Source: eClinicalMedicine. 2026 Oct 29;101:104302. doi: 10.1016/j.eclinm.2026.104302 (PMC13742331; doi:10.1016/j.eclinm.2026.104302)
Supplement: Supplementary file 1 [file mmc1.pdf]

SUPPLEMENTARY APPENDIX

Early Best Available Antibiotic Therapy for Multidrug-Resistant Gram-Negative Bloodstream Infections: A Target Trial Emulation

Luca Mezzadri, Wei Cong Tan, Xinxin Hao, Joan Quah, Abi Manesh, Ya-Ting Chang, Anh T.K. Nguyen, Ying Ding, Suwatthiya Kitsaran, Hock Hin Chua, Sotharith Bory, Sasheela Ponnampalavanar, Siriluck Anunnatsiri, Mohd Zulfakar Mazlan, Azizullah Khan Dhiloo, Rongpong Plongla, Andrea Lay-Hoon Kwa, Syed Faisal Mahmood, Arthur Dessi Roman, C Louise Thwaites, Phan Vinh Tho, Sheng Wu, Darunee Chotiprasitsakul, Ke Juin Wong, Hendri Wijaya, Cybele L. Abad, Muhammad Osama Rehman Khalid, Gazi Md. Salahuddin Mamun, Giri Shan Rajahram, Helio Gutierres, Huynh Ngoc Hon, Tran Van Giang, Ngoc Thach Pham, Abhilasha Karkey, Samanmalee Gunasekara, Raph L. Hamers, Robert Sinto, Inke N.D. Lubis, Minggui Wang, Merlin Moni, Po-Yu Liu, Yonghong Xiao, Basudha Khanal, N. Lakshmi Priya, Yohei Doi, Hitoshi Honda, Bayaraa Baljin, Ulziijargal Gurav, Rosmonaliza Asli, Lowell Ling, Ashesh Dhungana, Li-Yang Hsu, David L. Paterson, Yin Mo, *on behalf of ACORN-HAI investigators.*

Table of contents

Early Best Available Antibiotic Therapy for Multidrug-Resistant Gram-Negative Bloodstream Infections: A Target Trial Emulation 1

Supplementary Tables 3

Table S1. Hospital infrastructure of 41 study sites in ACORN-HAI study 3

Table S2. Specification of the target trial and its emulation. 4

Table S3. Definition of phenotypic resistance profile of bacterial pathogens in ACORN-HAI study 5

Table S4. Antibiotics considered for AST-based appropriateness definition in sensitivity analyses 6

Table S5. Baseline characteristics, by treatment group. 7

Table S6. Carbapenem-based regimens among patients with 3GCRE bloodstream infections receiving early BAT. .. 8

Table S7. Antibacterial therapy duration overall and across pathogen groups, stratified by treatment strategy. 9

Table S8. Crude mortality estimates overall and by pathogen group at 7, 14 and 28 days. 10

Table S9. Additive interaction between baseline severity (qSOFA ≥2) and absence of early best available therapy on 14-day mortality among patients with 3GCRE bloodstream infections. 11

Table S10. Additive interaction between baseline severity (qSOFA ≥2) and absence of early best available therapy on 14-day mortality among patients with CRE bloodstream infections. 12

Table S11. Additive interaction between baseline severity (qSOFA ≥2) and absence of early best available therapy on 14-day mortality among patients with CRA bloodstream infections. 13

Table S12. Additive interaction between baseline severity (Pitt bacteraemia score ≥3) and absence of early best available therapy on 14-day mortality among patients with 3GCRE bloodstream infections. 14

Table S13. Additive interaction between baseline severity (Pitt bacteraemia score ≥3) and absence of early best available therapy on 14-day mortality among patients with CRE bloodstream infections. 15

Table S14. Additive interaction between baseline severity (Pitt bacteraemia score ≥3) and absence of early best available therapy on 14-day mortality among patients with CRA bloodstream infections. 16

Table S15. IPTW-adjusted mortality estimates overall and by pathogen group at 7, 14, and 28 days in the sensitivity analysis defining early BAT as initiation on Day 0 only. 17

Table S16. IPTW-adjusted mortality estimates overall and by pathogen group at 7, 14, and 28 days in the sensitivity analysis defining early BAT as initiation on Day 1 only. 18

Table S17. IPTW-adjusted mortality estimates overall and by pathogen group at 7, 14, and 28 days using an AST-concordant BAT definition. 19

Table S18. IPTW-adjusted mortality estimates overall and by pathogen group at 7, 14, and 28 days using an in vitro activity-based definition of appropriate therapy. 20

Table S19. IPTW-adjusted mortality estimates overall and by pathogen group at 7, 14, and 28 days among patients who initiated BAT within one day of infection onset and continued therapy for at least three consecutive days. 21

Table S20. Comparison of truncated and untruncated stabilized inverse probability weights and corresponding effect estimates. 22

Supplementary Figures 23

Figure S1. Directed acyclic graph illustrating assumed causal relationships. 23

Figure S2. Covariate balance before and after inverse probability of treatment weighting, by pathogen group. 24

Figure S3. Distribution of best available antibiotic therapy days of therapy through Day 14. 25

Figure S4. Survival curves of crude 28-day survival according to early BAT overall and by pathogen group. 26

Supplementary Tables

Table S1. Hospital infrastructure of 41 study sites in ACORN-HAI study

	**Study sites (n = 41)**
**Participating countries**	
Bangladesh	1
Brunei	1
Cambodia	1
China	3
Hong Kong SAR	1
India	2
Indonesia	3
Japan	1
Malaysia	5
Mongolia	3
Nepal	3
Pakistan	3
the Philippines	2
Singapore	2
Sri Lanka	1
Taiwan	1
Thailand	4
Timor-Leste	1
Vietnam	3
**Number of hospital beds**	
< 500	10 (24%)
500 - 1000	13 (32%)
>1000	18 (44%)
**Number of ICU beds**	
< 50	17 (42%)
50-100	16 (39%)
>100	6 (15%)
Missing	2 (4%)
**Microbiology laboratory**	
CLSI guideline	35 (86%)
EUCAST guideline	4 (10%)
Missing	2 (4%)

Abbreviations: CLSI: Clinical and Laboratory Standards Institute; EUCAST: European Committee on Antimicrobial Susceptibility Testing; SAR, Special Administrative Region.

Table S2. Specification of the target trial and its emulation.

Protocol item	Target trial	Target trial emulation
**Eligibility**	Adults (≥18 years) hospitalized with hospital-acquired or healthcare-associated monomicrobial bloodstream infection due to CRE, 3GCR-E, or CRA, with the causative organism and resistance profile identified on the day of infection onset (Day 0).	Adults enrolled in ACORN-HAI with a first episode of hospital-acquired or healthcare-associated monomicrobial BSI due to CRE, 3GCR-E, or CRA, identified using phenotypic resistance criteria. Patients who died or were discharged within one calendar day of Day 0 were excluded because they could not plausibly initiate treatment within the exposure window.
**Treatment strategies**	Eligible patients would be assigned to either: (i) initiation on Day 0 of the best available guideline-recommended antibiotic regimen for the identified resistance profile, selected according to local drug availability; or (ii) no initiation of such regimen on Day 0, with subsequent therapy allowed according to routine care.	Antibiotic exposure was ascertained from prospectively collected treatment data. Best available therapy (BAT) was retrospectively classified according to a prespecified phenotype-specific definition based on international guidelines and local drug availability. In the primary analysis, initiation of BAT was operationalized as receipt of BAT on Day 0, allowing a ±1-day grace period.
**Assignment procedures**	Eligible patients would be randomly assigned at Day 0 to one of the two treatment strategies. Clinicians and patients would be aware of treatment allocation.	Treatment strategies were not randomly assigned. Stabilized inverse probability of treatment weights derived from propensity scores were used to adjust for measured baseline confounders and emulate random assignment conditional on these covariates.
**Follow-up**	Follow-up would begin at Day 0 and continue until Day 28 after infection onset.	Follow-up began at Day 0 and continued until Day 28 after infection onset, with administrative censoring at Day 28.
**Outcomes**	Primary outcome: 14-day all-cause mortality. Secondary outcomes: 7- and 28-day all-cause mortality.	Same as target trial. Outcomes were ascertained using prospectively collected ACORN-HAI follow-up data.
**Causal contrast**	Primary estimand: intention-to-treat effect of assignment to initiation versus non-initiation of the best available guideline-recommended antibiotic regimen on Day 0.<br><br>Secondary estimand: per-protocol effect of initiating BAT on Day 0 and adhering to the assigned strategy for at least 3 days.	Primary analysis: intention-to-treat-like effect comparing initiation versus non-initiation of BAT on Day 0, regardless of subsequent treatment modifications.<br><br>Sensitivity analysis: approximation of the per-protocol effect using a Day 4 landmark analysis, defining early BAT as initiation on Day 0 (±1 day) with continuation for at least three consecutive days.
**Identifying assumptions**	Exchangeability ensured by randomization; positivity; consistency.	Conditional exchangeability given measured baseline covariates at Day 0; positivity; consistency; and correct model specification. Covariates sufficient to satisfy exchangeability were selected a priori using a directed acyclic graph (Figure S1).
**Data-analysis plan**	Pre-specified statistical analysis plan, including interaction and sensitivity analyses.	Propensity scores were estimated using logistic regression including prespecified baseline covariates. Stabilized IPTW were applied to emulate random assignment and estimate weighted mortality risks. Weight diagnostics, covariate balance assessment, interaction analyses, and sensitivity analyses were prespecified.

Abbreviations: BSI, bloodstream infection; CRE, carbapenem-resistant Enterobacterales; 3GCRE, third-generation cephalosporin-resistant Enterobacterales; CRA, carbapenem-resistant Acinetobacter spp.; BAT, best available therapy; IPTW, inverse probability of treatment weighting.

Table S3. Definition of phenotypic resistance profile of bacterial pathogens in ACORN-HAI study

Phenotype name	Phenotype code	Phenotype definition
**Carbapenem-resistant *Acinetobacter* spp.**	CRA	Any *Acinetobacter* spp. that has tested Resistant (R) to at least one of the following: imipenem, meropenem, or doripenem.
**Carbapenem-resistant Enterobacterales**	CRE	Any Enterobacterales isolates that have tested Resistant (R) to at least one of the following: imipenem, meropenem, doripenem or ertapenem.
**Third-generation cephalosporin resistant Enterobacterales**	3GCRE	Any Enterobacterales isolates that have tested Resistant (R) to at least one of the following: cefixime, cefotaxime, ceftazidime or ceftriaxone, and is not classified as CRE.

Table S4. Antibiotics considered for AST-based appropriateness definition in sensitivity analyses

**Pathogen group**	**Antibiotics included in AST-based appropriateness definition**
**Third-generation cephalosporin-resistant Enterobacterales**	Ceftazidime-avibactam; Ceftolozane-tazobactam; Fosfomycin; Aztreonam; Meropenem; Imipenem; Ertapenem; Piperacillin-tazobactam; Levofloxacin; Ciprofloxacin; Amikacin; Gentamicin
**Carbapenem-resistant Enterobacterales**	Ceftazidime-avibactam; Colistin; Polymyxin B; Ceftolozane-tazobactam; Fosfomycin; Aztreonam; Amikacin; Gentamicin
**Carbapenem-resistant *Acinetobacter* spp.**	Ampicillin-sulbactam; Colistin; Polymyxin B; Fosfomycin; Cefoperazone-sulbactam; Amikacin; Gentamicin

Abbreviations: AST, antimicrobial susceptibility testing.

Table S5. Baseline characteristics, by treatment group.

Characteristic	No early BAT (N = 889)	Early BAT (N = 523)
Age (years), mean (SD)	59.0 (16.4)	59.5 (16.9)
Male sex, n (%)	491 (55%)	295 (56%)
Country income level, n (%)		
High income	73 (8.2%)	54 (10%)
Lower middle income	369 (42%)	181 (35%)
Upper middle income	447 (50%)	288 (55%)
Charlson Comorbidity Index, median (IQR)	1 (0-2)	1 (0-2)
Quick SOFA, median (IQR)	1 (0-2)	1 (0-2)
Pitt Bacteremia Score, median (IQR)	1 (0-3)	1 (0-4)
Organism, n (%)		
Acinetobacter spp.	179 (20%)	85 (16%)
E. coli	320 (36%)	219 (42%)
Klebsiella spp.	314 (35%)	184 (35%)
Enterobacter spp.	35 (3.9%)	22 (4.2%)
Other Enterobacterales	41 (4.6%)	13 (2.5%)
Length of stay before infection onset (days), median (IQR)	8 (2-16)	9 (1-18)
Admission ward type, n (%)		
General ward	591 (66%)	320 (61%)
ICU	298 (34%)	203 (39%)
ICU at infection onset, n (%)	320 (36%)	238 (46%)

Abbreviations: IQR = Interquartile range; ICU = Intensive Care Unit; BAT = Best Available Therapy; SD: standard deviation; SOFA, Sequential Organ Failure Assessment. Other Enterobacterales included *Citrobacter* spp. (n=12), *Proteus* spp. (n=21), *Serratia* spp. (n=10), *Morganella* spp. (n=5), *Raoultella* spp. (n=3), *Edwardsiella* spp. (n=1), *Pantoea* spp. (n=1), *Providencia* spp. (n=1).

Table S6. Carbapenem-based regimens among patients with 3GCRE bloodstream infections receiving early BAT.

Regimen	n (%)
**Type of carbapenem received (N=343)**	
Meropenem	310 (90.4%)
Ertapenem	20 (5.8%)
Imipenem	11 (3.2%)
Multiple carbapenems	2 (0.6%)
**Concomitant anti-Gram-negative therapy (N=343)**	
No additional anti-Gram-negative agent	165 (48.1%)
≥1 additional anti-Gram-negative agent	178 (51.9%)
**Additional anti-Gram-negative agents (N=178)**	
Piperacillin-tazobactam	86 (48.3%)
Ceftriaxone	34 (19.1%)
Amikacin	27 (15.2%)
Trimethoprim-sulfamethoxazole	15 (8.4%)
Ciprofloxacin	9 (5.1%)
Cefoperazone-sulbactam	8 (4.5%)
Levofloxacin	8 (4.5%)
Ceftazidime	7 (3.9%)
Amoxicillin-clavulanate	6 (3.4%)
Colistin	5 (2.8%)
Moxifloxacin	4 (2.2%)
Cefazolin	3 (1.7%)
Gentamicin	2 (1.1%)
Ampicillin-sulbactam	1 (0.6%)
Aztreonam	1 (0.6%)
Cefepime	1 (0.6%)
Cefixime	1 (0.6%)
Cefotaxime	1 (0.6%)
Cefuroxime	1 (0.6%)
Minocycline	1 (0.6%)

Note: Percentages for carbapenem type and concomitant anti-Gram-negative therapy were calculated among the 343 patients receiving carbapenem-based early BAT. Percentages for individual additional agents were calculated among the 178 patients receiving at least one additional anti-Gram-negative agent. Patients could receive more than one additional anti-Gram-negative agent; therefore, these categories are not mutually exclusive and percentages do not sum to 100%.

Additional anti-Gram-negative agents included any such agent administered on the same calendar day as the carbapenem; therefore, this category may include sequential administration during treatment transitions (e.g., piperacillin-tazobactam followed by meropenem)

Table S7. Antibacterial therapy duration overall and across pathogen groups, stratified by treatment strategy.

Pathogen group	Treatment strategy	Total antibacterial therapy, median (IQR), days	BAT among recipients, median (IQR), days
**Overall**	Early BAT	14 (8-20)	10 (6-15)
	No early BAT	11 (7-17)	8 (6-14)
**3GCRE**	Early BAT	13 (8-19)	10 (7-15)
	No early BAT	11 (7-16)	8 (5-11)
**CRE**	Early BAT	12 (7-18)	8 (5-15)
	No early BAT	11 (6-17)	9 (5-15)
**CRA**	Early BAT	16 (10-26)	11 (7-17)
	No early BAT	11 (7-17)	9 (6-14)

Note: Antibacterial therapy duration was defined as the number of days with antibacterial therapy from the day before BSI onset until hospital discharge or in-hospital death.

Abbreviations: BAT, best available therapy; IQR, interquartile range; BSI, bloodstream infection; 3GCRE, third-generation cephalosporin-resistant Enterobacterales; CRE, carbapenem-resistant Enterobacterales; CRA, carbapenem-resistant Acinetobacter spp.

Table S8. Crude mortality estimates overall and by pathogen group at 7, 14 and 28 days.

**Time-point**	**Pathogen group**	**No early BAT**	**Early BAT**	**p-value**
7 days	Overall	137/889 (15.4%)	65/523 (12.4%)	0.142
7 days	3GCRE	32/363 (8.8%)	22/343 (6.4%)	0.290
7 days	CRE	70/347 (20.2%)	32/95 (33.7%)	0.008
7 days	CRA	35/179 (19.6%)	11/85 (12.9%)	0.250
14 days	Overall	216/889 (24.3%)	109/523 (20.8%)	0.154
14 days	3GCRE	62/363 (17.1%)	38/343 (11.1%)	0.029
14 days	CRE	96/347 (27.7%)	46/95 (48.4%)	<0.001
14 days	CRA	58/179 (32.4%)	25/85 (29.4%)	0.728
28 days	Overall	283/889 (31.8%)	158/523 (30.2%)	0.565
28 days	3GCRE	85/363 (23.4%)	66/343 (19.2%)	0.208
28 days	CRE	121/347 (34.9%)	57/95 (60.0%)	<0.001
28 days	CRA	77/179 (43.0%)	35/85 (41.2%)	0.881

Abbreviations: BAT, best available antibiotic therapy; 3GCRE, third-generation cephalosporin-resistant Enterobacterales; CRE, carbapenem-resistant Enterobacterales; CRA, carbapenem-resistant Acinetobacter spp.
P values were calculated using Pearson's chi-square test.

Table S9. Additive interaction between baseline severity (qSOFA ≥2) and absence of early best available therapy on 14-day mortality among patients with 3GCRE bloodstream infections.

*	Early BAT	No early BAT	Effect of no early BAT within qSOFA strata
	RR [95% CI]	RR [95% CI]	RR [95% CI]
qSOFA <2	1 [Reference]	1.3 [0.74, 2.25]	1.3 [0.74, 2.25]
qSOFA ≥2	1.68 [0.89, 3.18]	4.03 [2.43, 6.69]	2.4 [1.4, 4.14]
Effect of qSOFA ≥2 within early BAT strata	1.68 [0.89, 3.18]	3.1 [1.99, 4.88]	
Multiplicative scale	1.85 [0.8, 4.03]		
RERI	2.06 [0.47, 4.41]		
AP	0.51 [0.14, 0.75]		
SI	3.11 [0.89, 10.79]		

Abbreviations: BAT, best available antibiotic therapy; qSOFA, quick Sequential Organ Failure Assessment; RR, risk ratio; CI, confidence interval; RERI, relative excess risk due to interaction; AP, attributable proportion due to interaction; SI, synergy index.

Table S10. Additive interaction between baseline severity (qSOFA ≥2) and absence of early best available therapy on 14-day mortality among patients with CRE bloodstream infections.

*	Early BAT	No early BAT	Effect of no early BAT within qSOFA strata
	RR [95% CI]	RR [95% CI]	RR [95% CI]
qSOFA <2	1 [Reference]	1.48 [0.7, 2.98]	1.48 [0.7, 2.98]
qSOFA ≥2	3.12 [1.52, 6.42]	2.68 [1.35, 5.34]	0.86 [0.6, 1.23]
Effect of qSOFA ≥2 within early BAT strata	3.12 [1.52, 6.42]	1.81 [1.32, 2.49]	
Multiplicative scale	0.58 [0.26, 1.28]		
RERI	-0.92 [-4.4, 0.21]		
AP	-0.34 [-0.95, 0.16]		
SI	0.65 [0.39, 1.08]		

Abbreviations: BAT, best available antibiotic therapy; qSOFA, quick Sequential Organ Failure Assessment; RR, risk ratio; CI, confidence interval; RERI, relative excess risk due to interaction; AP, attributable proportion due to interaction; SI, synergy index.

Table S11. Additive interaction between baseline severity (qSOFA ≥2) and absence of early best available therapy on 14-day mortality among patients with CRA bloodstream infections.

*	Early BAT	No early BAT	Effect of no early BAT within qSOFA strata
	RR [95% CI]	RR [95% CI]	RR [95% CI]
qSOFA <2	1 [Reference]	1.49 [0.64, 3.51]	1.49 [0.64, 3.51]
qSOFA ≥2	2.04 [0.86, 4.89]	2.48 [1.09, 5.63]	1.21 [0.76, 1.94]
Effect of qSOFA ≥2 within early BAT strata	2.04 [0.86, 4.89]	1.66 [1.08, 2.56]	
Multiplicative scale	0.81 [0.31, 2.15]		
RERI	-0.06 [-3.5, 1.58]		
AP	-0.02 [-0.69, 0.57]		
SI	0.96 [0.39, 2.36]		

Abbreviations: BAT, best available antibiotic therapy; qSOFA, quick Sequential Organ Failure Assessment; RR, risk ratio; CI, confidence interval; RERI, relative excess risk due to interaction; AP, attributable proportion due to interaction; SI, synergy index.

Table S12. Additive interaction between baseline severity (Pitt bacteraemia score ≥3) and absence of early best available therapy on 14-day mortality among patients with 3GCRE bloodstream infections.

*	Early BAT	No early BAT	Effect of no early BAT within Pitt score strata
	RR [95% CI]	RR [95% CI]	RR [95% CI]
Pitt score <3	1 [Reference]	1.52 [0.90, 2.58]	1.52 [0.90, 2.58]
Pitt score ≥3	2.87 [1.54, 5.35]	5.88 [3.55, 9.73]	2.05 [1.20, 3.50]
Effect of Pitt score ≥3 within early BAT strata	2.87 [1.54, 5.35]	3.86 [2.52, 5.92]	
Multiplicative scale	1.34 [0.63, 2.86]		
RERI	2.48 [0.09, 5.47]		
AP	0.42 [0.03, 0.65]		
SI	2.04 [0.98, 4.21]		

Abbreviations: BAT, best available antibiotic therapy; RR, risk ratio; CI, confidence interval; RERI, relative excess risk due to interaction; AP, attributable proportion due to interaction; SI, synergy index.

Table S13. Additive interaction between baseline severity (Pitt bacteraemia score ≥3) and absence of early best available therapy on 14-day mortality among patients with CRE bloodstream infections.

*	Early BAT	No early BAT	Effect of no early BAT within Pitt score strata
	RR [95% CI]	RR [95% CI]	RR [95% CI]
Pitt score <3	1 [Reference]	1.61 [0.81, 3.2]	1.61 [0.81, 3.2]
Pitt score ≥3	3.39 [1.67, 6.84]	2.86 [1.44, 5.68]	0.84 [0.59, 1.21]
Effect of Pitt score ≥3 within early BAT strata	3.39 [1.67, 6.84]	1.77 [1.29, 2.43]	
Multiplicative scale	0.52 [0.24, 1.13]		
RERI	-1.14 [-4.82, 0.09]		
AP	-0.4 [-1.02, 0.09]		
SI	0.62 [0.38, 1.01]		

Abbreviations: BAT, best available antibiotic therapy; RR, risk ratio; CI, confidence interval; RERI, relative excess risk due to interaction; AP, attributable proportion due to interaction; SI, synergy index.

Table S14. Additive interaction between baseline severity (Pitt bacteraemia score ≥3) and absence of early best available therapy on 14-day mortality among patients with CRA bloodstream infections.

*	Early BAT	No early BAT	Effect of no early BAT within Pitt score strata
	RR [95% CI]	RR [95% CI]	RR [95% CI]
Pitt score <3	1 [Reference]	1.57 [0.68, 3.63]	1.57 [0.68, 3.63]
Pitt score ≥3	2.29 [0.97, 5.41]	2.76 [1.22, 6.20]	1.20 [0.75, 1.92]
Effect of Pitt score ≥3 within early BAT strata	2.29 [0.97, 5.41]	1.75 [1.15, 2.68]	
Multiplicative scale	0.77 [0.29, 2.00]		
RERI	-0.10 [-3.75, 1.71]		
AP	-0.04 [-0.68, 0.51]		
SI	0.94 [0.43, 2.09]		

Abbreviations: BAT, best available antibiotic therapy; RR, risk ratio; CI, confidence interval; RERI, relative excess risk due to interaction; AP, attributable proportion due to interaction; SI, synergy index.

Table S15. IPTW-adjusted mortality estimates overall and by pathogen group at 7, 14, and 28 days in the sensitivity analysis defining early BAT as initiation on Day 0 only.

**Pathogen group**	**Outcome**	**Expected mortality risk for early BAT (95% CI)**	**Expected mortality risk for no early BAT (95% CI)**	**RD (95% CI)**
**Overall (N = 1511)**	7-day mortality	13.8% (10.4-17.3)	20.9% (18.4-23.4)	-7.1% (-11.1, -2.7)
	14-day mortality	21.3% (17.4-25.5)	29.1% (26.4-31.8)	-7.8% (-12.5, -2.4)
	28-day mortality	30.7% (26.1-35.7)	36.5% (33.6-39.1)	-5.7% (-10.9, 0.4)
**3GCR-E (N = 735)**	7-day mortality	8.1% (4.9-11.6)	11.5% (8.6-14.7)	-3.4% (-8.0, 1.2)
	14-day mortality	12.3% (8.5-16.7)	18.3% (14.9-22.0)	-6.0% (-11.2, -0.8)
	28-day mortality	19.4% (14.7-24.2)	25.3% (21.5-29.7)	-5.9% (-12.0, 0.4)
**CRE (N = 477)**	7-day mortality	23.4% (15.0-35.3)	28.5% (24.1-33.0)	-5.1% (-14.7, 7.9)
	14-day mortality	34.8% (23.1-50.1)	36.4% (31.6-41.0)	-1.6% (-14.2, 14.3)
	28-day mortality	49.0% (34.2-66.0)	42.9% (38.0-47.7)	6.1% (-9.8, 23.7)
**CRA (N = 299)**	7-day mortality	10.5% (3.6-20.7)	27.8% (22.2-33.6)	-17.3% (-26.0, -6.3)
	14-day mortality	33.6% (18.7-49.3)	39.3% (33.1-45.6)	-5.7% (-22.0, 10.2)
	28-day mortality	47.2% (31.7-63.2)	49.5% (43.2-55.2)	-2.2% (-19.2, 14.4)

Note: The larger sample size compared with the primary analysis reflects the Day 0-only treatment assignment window. Patients were not required to remain alive and hospitalised through Day 1; however, those who died or were discharged on Day 0 remained excluded to avoid immortal time bias.

Abbreviations: BAT, best available antibiotic therapy; RD, risk difference; CI, confidence interval; 3GCR-E, third-generation cephalosporin-resistant Enterobacterales; CRE, carbapenem-resistant Enterobacterales; CRA, carbapenem-resistant *Acinetobacter* spp.

Table S16. IPTW-adjusted mortality estimates overall and by pathogen group at 7, 14, and 28 days in the sensitivity analysis defining early BAT as initiation on Day 1 only.

**Pathogen group**	**Outcome**	**Expected mortality risk for early BAT (95% CI)**	**Expected mortality risk for no early BAT (95% CI)**	**RD (95% CI)**
**Overall (N = 1412)**	7-day mortality	11.2% (8.8-13.8)	16.3% (13.9-18.9)	-5.1% (-8.6, -1.4)
	14-day mortality	18.8% (15.6-21.9)	25.5% (22.6-28.6)	-6.8% (-11.1, -2.6)
	28-day mortality	27.8% (23.9-31.4)	33.2% (29.9-36.6)	-5.4% (-10.8, 0.7)
**3GCR-E (N = 706)**	7-day mortality	6.3% (3.7-9.2)	9.1% (6.4-12.3)	-2.9% (-6.9, 1.2)
	14-day mortality	10.0% (6.9-13.4)	17.2% (13.3-21.4)	-7.2% (-12.1, -2.2)
	28-day mortality	17.5% (13.3-21.9)	24.0% (19.7-28.5)	-6.5% (-12.6, -0.4)
**CRE (N = 442)**	7-day mortality	19.6% (11.2-31.0)	22.6% (17.8-27.5)	-3.0% (-12.3, 9.5)
	14-day mortality	28.0% (18.6-40.8)	30.6% (25.4-36.1)	-2.6% (-13.0, 11.3)
	28-day mortality	34.8% (24.2-49.9)	38.5% (32.3-44.1)	-3.7% (-14.9, 12.4)
**CRA (N = 264)**	7-day mortality	11.8% (5.3-18.9)	19.7% (13.8-26.2)	-7.9% (-17.6, 1.0)
	14-day mortality	25.1% (16.2-35.2)	32.9% (25.5-40.6)	-7.7% (-19.0, 3.9)
	28-day mortality	37.4% (26.6-48.7)	45.0% (36.8-52.9)	-7.6% (-20.9, 6.4)

Abbreviations: BAT, best available antibiotic therapy; RD, risk difference; CI, confidence interval; 3GCR-E, third-generation cephalosporin-resistant Enterobacterales; CRE, carbapenem-resistant Enterobacterales; CRA, carbapenem-resistant *Acinetobacter* spp.

Table S17. IPTW-adjusted mortality estimates overall and by pathogen group at 7, 14, and 28 days using an AST-concordant BAT definition.

Pathogen group	Outcome	Expected mortality risk for early BAT (95% CI)	Expected mortality risk for no early BAT (95% CI)	RD (95% CI)
**Overall (N = 1,319)**	7-day mortality	10.2% (7.0-13.3)	16.4% (13.9-18.7)	-6.2% (-10.1, -2.2)
	14-day mortality	16.5% (12.9-20.5)	25.9% (23.1-28.7)	-9.5% (-13.9, -4.9)
	28-day mortality	25.2% (21.1-29.8)	33.5% (30.5-36.5)	-8.3% (-13.1, -3.0)
**3GCR-E (N = 667)**	7-day mortality	6.0% (3.6-8.8)	9.2% (6.4-12.5)	-3.2% (-7.1, 0.6)
	14-day mortality	9.8% (6.7-13.2)	17.3% (13.5-21.8)	-7.6% (-12.7, -2.4)
	28-day mortality	16.6% (12.2-21.1)	24.2% (19.7-28.9)	-7.6% (-13.7, -1.2)
**CRE (N = 414)**	7-day mortality	18.8% (6.8-33.1)	23.2% (18.8-28.0)	-4.4% (-17.3, 10.3)
	14-day mortality	28.5% (12.7-45.3)	32.4% (27.7-37.6)	-3.9% (-20.4, 14.2)
	28-day mortality	40.9% (23.6-59.3)	39.8% (34.7-45.2)	1.1% (-18.2, 19.5)
**CRA (N = 238)**	7-day mortality	16.1% (6.2-27.5)	18.7% (13.6-24.6)	-2.5% (-14.3, 11.0)
	14-day mortality	27.8% (15.1-42.8)	31.5% (24.9-38.1)	-3.7% (-17.6, 11.9)
	28-day mortality	40.0% (24.1-56.5)	42.8% (35.1-50.0)	-2.8% (-19.3, 15.0)

Footnote: AST-concordant BAT was defined as receipt of a BAT agent with available *in vitro* susceptibility testing indicating susceptible or intermediate susceptibility. Patients who received early BAT for which AST results were unavailable were excluded from this sensitivity analysis, resulting in a smaller sample size compared with the primary analysis. The numbers of patients receiving early AST-concordant BAT versus comparator were 400 versus 919 overall, 304 versus 363 for 3GCR-E, 47 versus 367 for CRE, and 49 versus 189 for CRA, respectively.

Abbreviations: BAT, best available antibiotic therapy; RD, risk difference; CI, confidence interval; 3GCR-E, third-generation cephalosporin-resistant Enterobacterales; CRE, carbapenem-resistant Enterobacterales; CRA, carbapenem-resistant *Acinetobacter* spp.

Table S18. IPTW-adjusted mortality estimates overall and by pathogen group at 7, 14, and 28 days using an in vitro activity-based definition of appropriate therapy.

Pathogen group	Outcome	Expected mortality risk for early appropriate therapy (95% CI)	Expected mortality risk for no early appropriate therapy (95% CI)	RD (95% CI)
**Overall (N=1280)**	7-day mortality	11.3% (8.8, 14.2)	16.3% (13.7, 18.8)	-4.9% (-8.6, -1.0)
	14-day mortality	17.6% (14.5, 20.8)	26.4% (23.1, 29.7)	-8.8% (-13.5, -4.5)
	28-day mortality	26.8% (23.0, 30.6)	34.1% (30.6, 37.6)	-7.3% (-12.3, -2.4)
**3GCRE (N=636)**	7-day mortality	7.8% (5.4, 10.1)	7.6% (3.8, 11.8)	0.2% (-4.6, 4.7)
	14-day mortality	11.7% (8.9, 14.7)	17.4% (12.1, 23.5)	-5.7% (-12.7, 0.4)
	28-day mortality	18.8% (15.0, 22.3)	24.2% (17.7, 30.7)	-5.4% (-12.4, 1.9)
**CRE (N=409)**	7-day mortality	18.7% (9.1, 29.0)	23.4% (19.2, 28.3)	-4.6% (-15.3, 6.6)
	14-day mortality	27.2% (16.4, 40.0)	32.0% (27.1, 37.1)	-4.7% (-17.0, 8.6)
	28-day mortality	40.5% (27.6, 55.0)	39.4% (34.2, 44.9)	1.1% (-13.3, 16.0)
**CRA (N=235)**	7-day mortality	14.3% (5.5, 24.6)	18.1% (12.7, 24.1)	-3.8% (-14.0, 7.9)
	14-day mortality	25.9% (14.9, 38.4)	31.1% (24.6, 38.3)	-5.3% (-18.2, 9.0)
	28-day mortality	36.8% (23.6, 50.5)	42.6% (35.0, 49.9)	-5.8% (-20.4, 9.5)

Footnote: Appropriate therapy was defined as receipt of a clinically plausible antibiotic for the corresponding pathogen (Table S4) with available *in vitro* susceptibility indicating intermediate or susceptible. Patients who received an eligible antibiotic during the early treatment window but for whom susceptibility to all eligible agents received could not be determined were excluded from this sensitivity analysis. Consequently, the analytic sample was smaller than in the primary analysis. The numbers of patients receiving early in vitro-active therapy versus comparator were 575 versus 705 overall, 453 versus 183 for 3GCRE, 71 versus 338 for CRE, and 51 versus 184 for CRA, respectively.

Abbreviations: BAT, best available antibiotic therapy; RD, risk difference; CI, confidence interval; 3GCR-E, third-generation cephalosporin-resistant Enterobacterales; CRE, carbapenem-resistant Enterobacterales; CRA, carbapenem-resistant *Acinetobacter* spp.

Table S19. IPTW-adjusted mortality estimates overall and by pathogen group at 7, 14, and 28 days among patients who initiated BAT within one day of infection onset and continued therapy for at least three consecutive days.

**Pathogen group**	**Outcome**	**Expected mortality risk for early BAT (95% CI)**	**Expected mortality risk for no early BAT (95% CI)**	**RD (95% CI)**
**Overall (N = 1277)**	7-day mortality	6.0% (3.9-8.4)	10.6% (8.5-12.7)	-4.6% (-7.7, -1.2)
	14-day mortality	13.5% (10.5-16.5)	21.1% (18.4-24.0)	-7.6% (-11.7, -3.6)
	28-day mortality	22.6% (19.1-27.0)	29.8% (26.7-32.8)	-7.2% (-12.2, -1.9)
**3GCR-E (N = 650)**	7-day mortality	2.9% (1.0-5.2)	5.3% (3.0-8.0)	-2.4% (-5.7, 0.8)
	14-day mortality	6.9% (4.1-9.9)	13.7% (10.0-17.5)	-6.9% (-11.9, -2.1)
	28-day mortality	14.4% (10.2-18.7)	21.4% (17.2-26.0)	-7.0% (-13.1, -0.7)
**CRE (N = 392)**	7-day mortality	17.8% (8.1-28.6)	15.3% (11.6-19.5)	2.5% (-8.0, 14.0)
	14-day mortality	25.0% (14.5-36.7)	25.6% (21.1-30.7)	-0.6% (-12.3, 12.0)
	28-day mortality	33.3% (22.0-46.7)	34.2% (28.9-39.5)	-0.9% (-13.6, 14.0)
**CRA (N = 235)**	7-day mortality	5.6% (0.8-12.0)	12.8% (8.0-18.2)	-7.2% (-14.9, 0.8)
	14-day mortality	19.3% (10.6-29.5)	27.9% (21.1-34.9)	-8.6% (-19.8, 3.5)
	28-day mortality	31.9% (20.3-45.0)	41.7% (34.3-49.9)	-9.9% (-24.2, 5.0)

Note: Numbers shown in brackets reflect the modified treatment assignment window and corresponding eligibility criteria.

Abbreviations: BAT, best available antibiotic therapy; RD, risk difference; CI, confidence interval; 3GCR-E, third-generation cephalosporin-resistant Enterobacterales; CRE, carbapenem-resistant Enterobacterales; CRA, carbapenem-resistant *Acinetobacter* spp.

Table S20. Comparison of truncated and untruncated stabilized inverse probability weights and corresponding effect estimates.

Cohort	Weight specification	Mean (SD)	Range	Weights modified, n (%)	7-day RD (95% CI)	14-day RD (95% CI)	28-day RD (95% CI)
**Overall (n=1412)**	Untruncated	1.000 (0.232)	0.540-2.38	-	-5.3% (-8.8 to -1.6)	-7.1% (-11.4 to -2.9)	-6.0% (-11.4 to -1.3)
	Truncated (1st-99th)	0.999 (0.224)	0.599-1.80	30 (2.1%)	-5.3% (-8.8 to -1.6)	-7.1% (-11.3 to -3.0)	-5.9% (-11.3 to -1.1)
**3GCRE (n=706)**	Untruncated	1.000 (0.328)	0.570-2.75	-	-3.0% (-7.0 to 1.0)	-7.4% (-12.2 to -2.4)	-6.7% (-12.8 to -0.7)
	Truncated (1st-99th)	0.998 (0.321)	0.596-2.19	16 (2.3%)	-3.0% (-7.0 to 0.9)	-7.4% (-12.2 to -2.5)	-6.7% (-12.9 to -0.7)
**CRE (n=442)**	Untruncated	1.020 (0.786)	0.252-9.16	-	-4.7% (-13.5 to 7.4)	-5.3% (-15.4 to 8.8)	-7.1% (-18.1 to 9.4)
	Truncated (1st-99th)	1.000 (0.606)	0.287-4.42	10 (2.3%)	-3.3% (-12.4 to 9.2)	-3.3% (-13.8 to 10.4)	-4.7% (-15.8 to 11.3)
**CRA (n=264)**	Untruncated	0.988 (0.338)	0.479-2.61	-	-7.9% (-17.6 to 1.3)	-7.8% (-19.3 to 3.5)	-8.4% (-21.2 to 5.3)
	Truncated (1st-99th)	0.984 (0.321)	0.500-2.04	6 (2.3%)	-7.8% (-17.5 to 1.6)	-7.5% (-19.1 to 4.0)	-7.9% (-20.8 to 5.9)

Abbreviations: RD, risk difference; 3GCRE, third-generation cephalosporin-resistant Enterobacterales; CRE, carbapenem-resistant Enterobacterales; CRA, carbapenem-resistant Acinetobacter spp.
Stabilized inverse probability weights were truncated at the 1st and 99th percentiles in the primary analysis. Effect estimates are expressed as percentage-point risk differences.

Supplementary Figures

Figure S1. Directed acyclic graph illustrating assumed causal relationships.

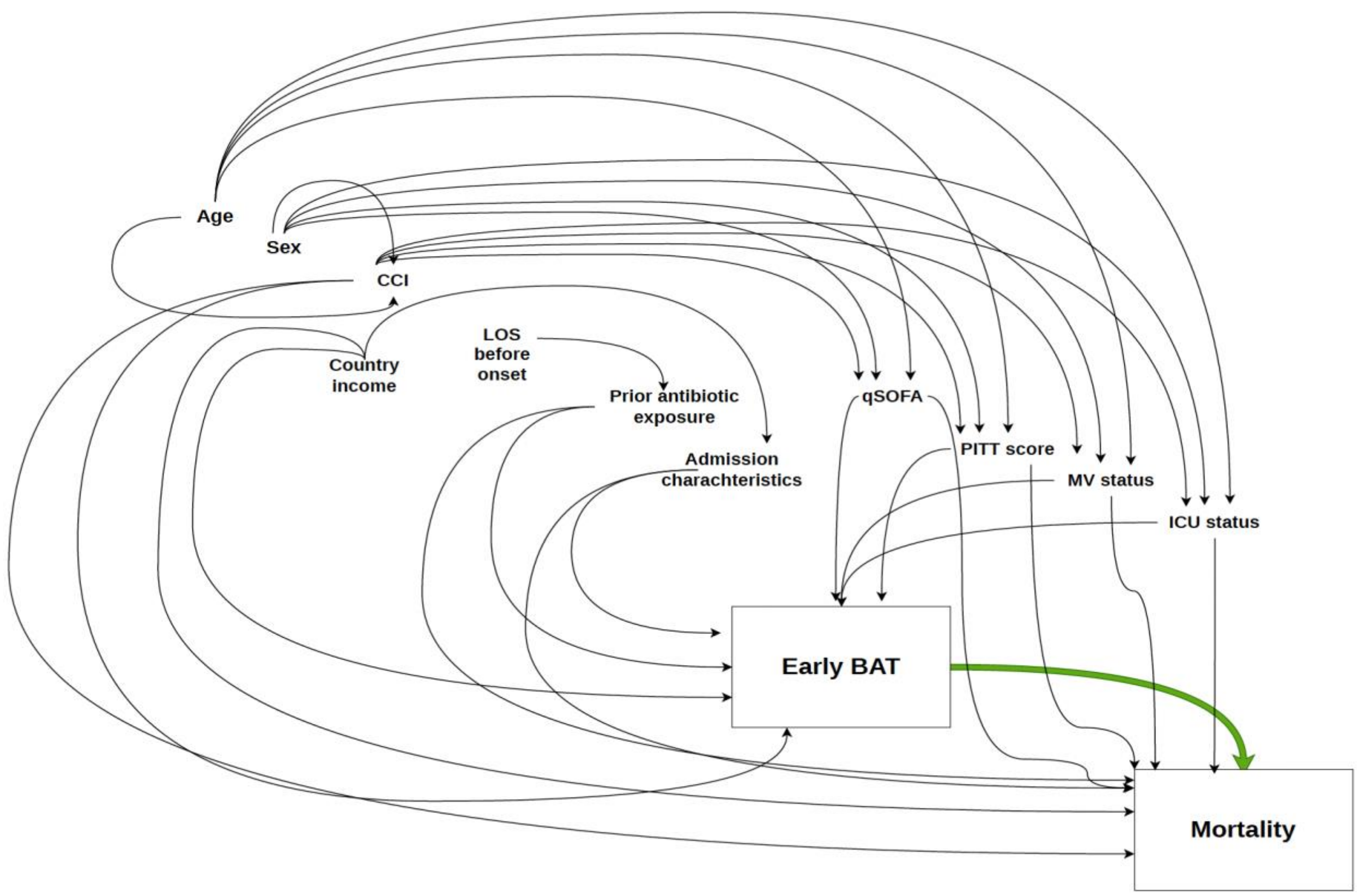

Abbreviations: qSOFA, quick Sequential Organ Failure Assessment; PITT, Pitt bacteremia score; ICU, intensive care unit; MV, mechanical ventilation; BAT, best available antibiotic therapy; LOS, length of stay; CCI, Charlson Comorbidity Index.

Figure S2. Covariate balance before and after inverse probability of treatment weighting, by pathogen group.

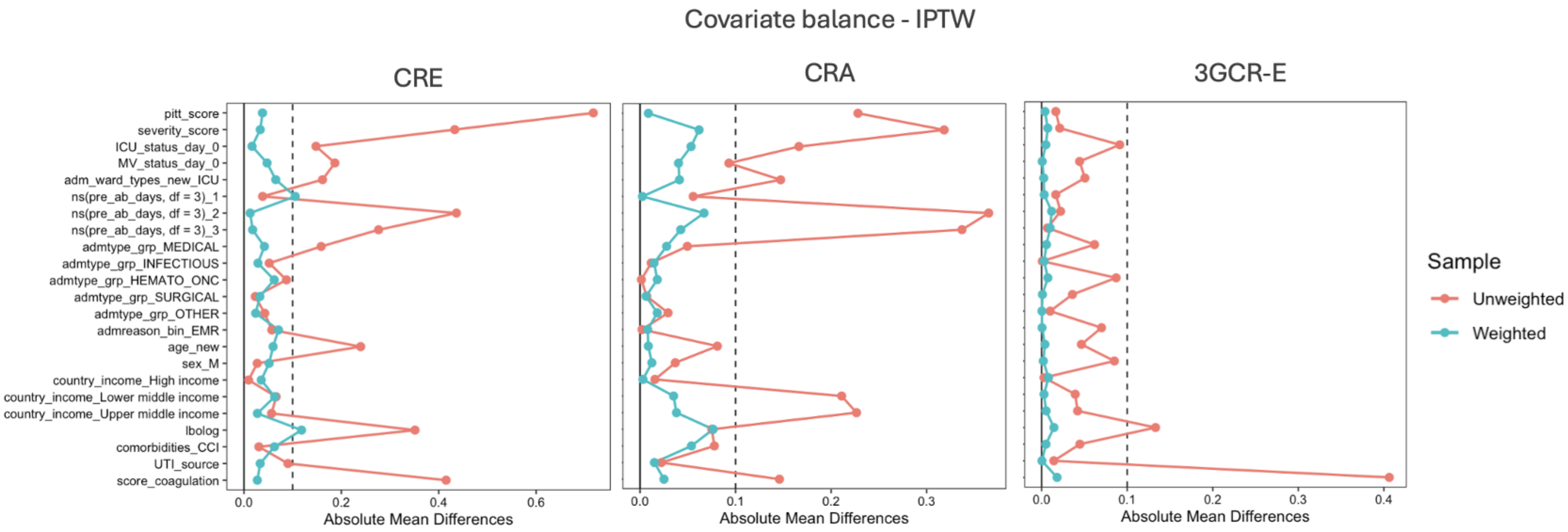

Abbreviations: CRE, carbapenem-resistant Enterobacterales; CRA, carbapenem-resistant Acinetobacter spp.; 3GCR-E, Third-generation cephalosporin resistant Enterobacterales; IPTW, inverse probability of treatment weighting.

Figure S3. Distribution of best available antibiotic therapy days of therapy through Day 14.

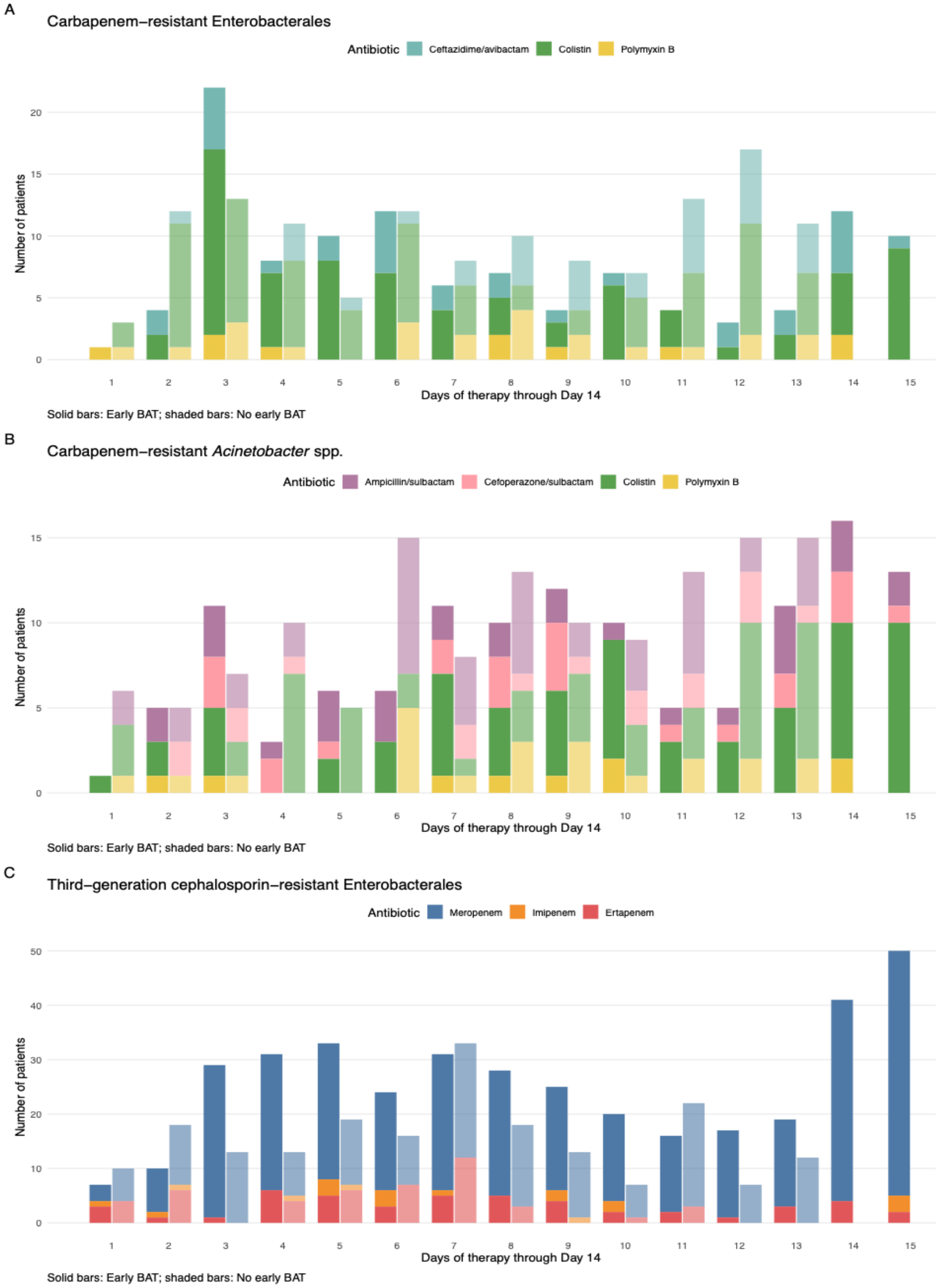

Note: Days of therapy (DOTs) were calculated from Day 0 through Day 14, death, or discharge, whichever occurred first. Bars for no early BAT are absent at 14-15 DOTs because patients reaching these durations had necessarily initiated BAT within the early-treatment window. Abbreviation: BAT, best available therapy.

Figure S4. Survival curves of crude 28-day survival according to early BAT overall and by pathogen group.

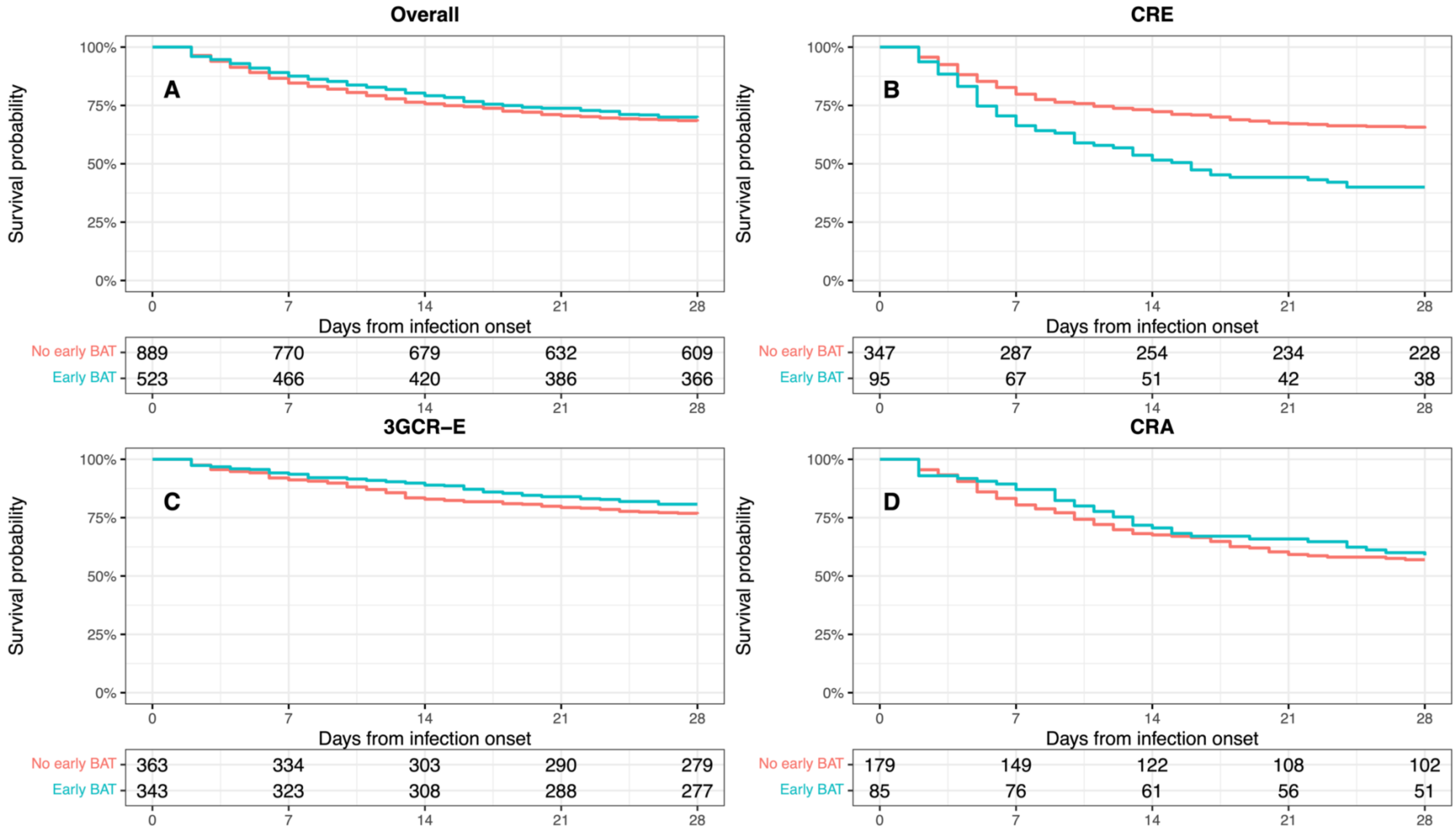

Abbreviations: CRE, carbapenem-resistant Enterobacterales; CRA, carbapenem-resistant *Acinetobacter* spp.; 3GCR-E, Third-generation cephalosporin resistant Enterobacterales; BAT, best available therapy
