# Supplementary material for: Early best available antibiotic therapy for multidrug-resistant gram-negative bloodstream infections: a target trial emulation
Source: eClinicalMedicine. 2026 Oct 29;101:104302. doi: 10.1016/j.eclinm.2026.104302 (PMC13742331; doi:10.1016/j.eclinm.2026.104302)
Supplement: Supplementary file 2 [file mmc2.docx]

**Group authorship**

| **First name** | **Last name** | **Affiliation** |
| --- | --- | --- |
| Jie | Su | ADVANCE-ID, Saw Swee Hock School of Public Health, National University of Singapore, Singapore |
| May Gee | Ong | ADVANCE-ID, Saw Swee Hock School of Public Health, National University of Singapore, Singapore |
| Kee Mee | Tan | ADVANCE-ID, Saw Swee Hock School of Public Health, National University of Singapore, Singapore |
| Amanda Wei Ping | Ling | ADVANCE-ID, Saw Swee Hock School of Public Health, National University of Singapore, Singapore |
| Shweta | Rajkumar Singh | ADVANCE-ID, Saw Swee Hock School of Public Health, National University of Singapore, Singapore |
| Siew Pei | Ong | ADVANCE-ID, Saw Swee Hock School of Public Health, National University of Singapore, Singapore |
| Jia Rhou | Lai | ADVANCE-ID, Saw Swee Hock School of Public Health, National University of Singapore, Singapore |
| Shiau Hui | Diong | National Centre for Infectious Diseases, Singapore |
| Suchart | Booraphun | Sunpasitthiprasong hospital, Ubon Ratchathani, Thailand |
| Ratiporn | Bansong | Sunpasitthiprasong hospital, Ubon Ratchathani, Thailand |
| Xun Ting | Tiong | Sarawak General Hospital, Malaysia |
| Alisa Hanum | Arba’eni | Sarawak General Hospital, Malaysia |
| Sophany | Koy | Calmette Hospital, Cambodia |
| Seanghuoy | Ho | Calmette Hospital, Cambodia |
| Helmi Bin | Sulaiman | University Malaya Medical Centra, Malaysia |
| Soo Nee | Tang | University Malaya Medical Centra, Malaysia |
| Pope | Kosalaraksa | Faculty of Medicine, Khon Kaen University, Thailand |
| Zeti Norfidiyati | Salmuna | School of Medical Sciences, Universiti Sains Malaysia, Malaysia |
| Gabriel Zherong | Yan | National University Hospital, Singapore |
| Ambreen | Fatima | Dow University of Health Sciences, Pakistan |
| Suvaporn | Anugulruengkitt | Chulalongkorn University and King Chulalongkorn Memorial Hospital, Thailand |
| Sadia | Shakoor | The Aga Khan University Hospital, Pakistan |
| Cecilia C. Maramba | Lazarte | Philippine General Hospital, Philippines |
| Thi Kim Anh | Nguyen | Hospital For Tropical Diseases, Ho Chi Minh City, Vietnam |
| Jinlan | Lin | Beijing Tsinghua Changgung Hospital, China |
| Pitak | Santanirand | Ramathibodi Hospital, Thailand |
| Ruth Tsin En | Cheng | Sabah Women and Children's Hospital, Malaysia |
| Rina | Yunita | General Hospital H. Adam Malik, Sumatera Utara, Indonesia |
| Nicole Bernadette | Terre | The Medical City, Philippines |
| Madiha | Hashmi | Dr. Ziauddin Hospital Clifton Campus, Pakistan |
| Md. Ali Amin | Nabin | International Centre for Diarrhoeal Disease Research, Bangladesh |
| Mohd Zaki Bin Mohd | Zaili | Queen Elizabeth II Hospital, Kota Kinabalu, Sabah, Malaysia |
| Joshua | Francis | Hospital Nacional Guido Valadares, Timor-Leste |
| H. Rogier van | Doorn | Oxford University Clinical Research Unit, 78 Giai Phong, Dong Da, Ha Noi, Vietnam National Hospital For Tropical Diseases, Ha Noi, Vietnam |
| Bidhya Sharma | Sharma | Patan Hospital, Kathmandu, Nepal |
| Sumudu | Withanage | National Cancer Institute, Sri Lanka |
| Yulia R. | Saharman | Cipto Mangunkusumo National Hospital, Faculty of Medicine Universitas Indonesia, Indonesia |
| R. Lia Kusumawati | Kusumawati | Universitas Sumatera Utara, Faculty of Medicine, Sumatera Utara, Indonesia |
| Xiaohua | Qin | Huashan Hospital Affiliated to Fudan University, China |
| Chithra | Tomy | Amrita Institute of Medical Sciences, Kochi, India |
| Chien-Hao | Tseng | Taichung Veteran General Hospital, Taiwan |
| Ping | Shen | The First Affiliated Hospital, Zhejiang University, China |
| Shyam Prasad | Kafle | B.P. Koirala Institute of Health Sciences, Nepal |
| T | Palaniappan | Medway Hospitals, India |
| Shogo | Hanai | Fujita Health University Hospital, Japan |
| Damdindorj | Boldbaatar | Mongolia-Japan Hospital of the Mongolian National University of Medical Sciences, Mongolia |
| Mohamed Ibrahim Ali | Alsaman | Raja Isteri Pengiran Anak Saleha Hospital, Brunei |
| Khurelbaatar | Nyamdavaa | Third State Central Hospital, Mongolia |
| Damdindorj | Boldbaatar | Central Military Hospital, Mongolia |
| Christopher | Lai | The Chinese University Of Hong Kong, Hong Kong, China |
| Santosh | Poudel | Bir Hospital, Kathmandu, Nepal |
